# A molybdenum oxide-based degradable nanosheet for combined chemo-photothermal therapy to improve tumor immunosuppression and suppress distant tumors and lung metastases

**DOI:** 10.1186/s12951-021-01162-2

**Published:** 2021-12-19

**Authors:** Na Qiu, Xiaoye Yang, Yanan Zhang, Jicheng Zhang, Jianbo Ji, Yu Zhang, Xinru Kong, Yanwei Xi, Dongzhu Liu, Lei Ye, Guangxi Zhai

**Affiliations:** 1 Department of Pharmaceutics, Key Laboratory of Chemical Biology (Ministry of Education), School of Pharmaceutical Sciences, Shandong University, 44 WenhuaXilu, Jinan, 250012 People’s Republic of China; 2 Department of Chemistry and Bioengineering, University of Washington, Seattle, WA 98195 USA

**Keywords:** Molybdenum oxide nanosheet, Photothermal and chemotherapy, Meliorate tumor immunosuppression, Distant tumor and lung metastasis

## Abstract

Molybdenum oxide (MoOx) nanosheets have drawn increasing attention for minimally invasive cancer treatments but still face great challenges, including complex modifications and the lack of efficient accumulation in tumor. In this work, a novel multifunctional degradable FA-BSA-PEG/MoOx nanosheet was fabricated (LA-PEG and FA-BSA dual modified MoOx): the synergistic effect of PEG and BSA endows the nanosheet with excellent stability and compatibility; the FA, a targeting ligand, facilitates the accumulation of nanosheets in the tumor. In addition, DTX, a model drug for breast cancer treatment, was loaded (76.49%, 1.5 times the carrier weight) in the nanosheets for in vitro and in vivo antitumor evaluation. The results revealed that the FA-BSA-PEG/MoOx@DTX nanosheets combined photothermal and chemotherapy could not only inhibit the primary tumor growth but also suppress the distant tumor growth (inhibition rate: 51.7%) and lung metastasis (inhibition rate: 93.6%), which is far more effective compared to the commercial Taxotere®. Exploration of the molecular mechanism showed that in vivo immune response induced an increase in positive immune responders, suppressed negative immune suppressors, and established an inflammatory tumor immune environment, which co-contributes towards effective suppression of tumor and lung metastasis. Our experiments demonstrated that this novel multifunctional nanosheet is a promising platform for combined chemo-photothermal therapy.

**Supplementary Information:**

The online version contains supplementary material available at 10.1186/s12951-021-01162-2.

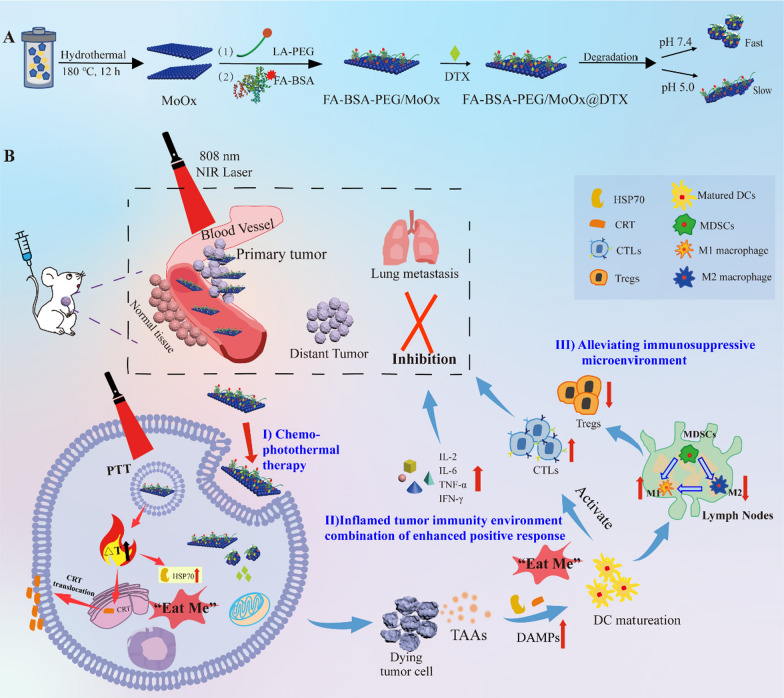

## Introduction

Even today, cancer remains a significant global health challenge [1]. Despite significant efforts in cancer treatment research over generations, strategies for clinical application remain limited due to the complexity and heterogeneity of tumors and the remarkable difference between in vitro and in vivo conditions [2, 3]. Conventional cancer treatments exhibit severe side effects, drug resistance, and limited effectiveness of monotherapy [4, 5]. Thus, the most promising strategy that overcomes all the above limitations is to fabricate a multifunctional nanocarrier combining multitherapy for the synergistic enhancement of cancer therapy.

Photothermal therapy (PTT) has gained increasing attention due to its effectiveness minimally invasive and high targeting properties. It utilizes photothermal agents to convert energy from a near-infrared (NIR) laser into heat and cause hyperthermal tumor damage, including protein denaturation and nucleic acid damage [6–8]. In addition, PTT has the potential to induce immunogenic cell death (ICD), release tumor-associated antigens (TAAs) and damage-associated molecular patterns (DAMPs), and activate the immune response, suggesting a promising approach for the combined chemo-photothermal therapy strategy [9, 10].

Among all the materials, the inorganic materials are attracting particular attention for cancer treatment with super high drug loading (could be as high as 2 times of the carrier weight), controllable size and PTT effect [11, 12]. However, unlike organic materials that are enzyme-degradable in our body, inorganic materials show difficulty in degradability and possess a risk of aggregation and accumulation in the body, leading to extreme toxicity. Therefore, the degradability and compatibility of the inorganic materials are the main factors that limited its application. The conventional strategy is to prepare ultrasmall nanosheets and modify them with a thin hydrophilic layer, so as to minimize their hydrodynamic sizes in the physiological environment to enable rapid renal filtration, avoid the accumulation in normal organs and thereby reduce the toxicity [13, 14]. However, these ultrasmall nanosheets would behave similar to small molecules: their rapid renal excretion would also shorten the blood circulation half-lives and reduce drug bioavailability, resulting in a reduced accumulation and antitumor effect. Therefore, the ideal inorganic carrier should not only be quickly excretable from the normal organs of the body to reduce toxicity, but also be capable of efficient accumulation and retention in the tumor to increase therapeutic efficacy.

Molybdenum oxide (MoOx) nanosheets show great potential for minimally invasive cancer treatment due to their high NIR absorbance and pH-dependent oxidative degradation properties, which are widely exploited in electrocatalysis, photocatalysis, chemical sensing, and biomedical fields [15–18]. Also, this photothermal agent could be taken as a drug delivery carrier for combination therapy, contributing to cancer treatment [19–21]. However, the strong hydrophobic interactions cause aggregation and precipitation, which has remarkably limited its application, especially in the biomedical field. As intravenous administration preparation, the complexity of the blood requires that the nanoparticles have extraordinary stability to resist protein adsorption and sustain stability for the tumor-targeted drug delivery [22, 23]. Though they have exhibited potential in various medical applications, the successful preparation of MoOx-based nanosheets with the combination of the above mentioned functions for cancer treatment, especially chemo-photothermal therapy, has not been reported.

To this end, we introduce here a super stability and targeting strategy to expand the application of the MoOx nanosheet in the cancer treatment field. First, α-lipoic acid conjugated mPEG-NH_2_ (LA-PEG) was applied for improving the solubility of MoOx nanosheets by the thiol group with the oxygen-deficient site on the surface of MoOx under ultrasound [24]. To further increase nanosheet stability and compatibility, the folic acid (FA) modified bovine serum albumin (FA-BSA) was introduced into the system, as a strong stabilizer to prevent the re-aggregation of single-layer nanosheets and improve biocompatibility. Moreover, the non-polar groups of the protein can be firmly combined with the MoOx layer with the hydrophilic groups exposed to water, promoting the stability of peeled nanosheets [25, 26]. The FA on the nanosheet surface could target tumor cells by the specific FA receptor, which is overexpressed on the surface of breast cancer cells. The dual modified MoOx (FA-BSA-PEG/MoOx) could significantly increase its stability, biocompatibility and targeting properties without compromising photothermal conversion efficiency (43.41%). Subsequently, the docetaxel (DTX), a chemotherapy agent for breast cancer treatment, was loaded on the nanosheet, and drug loading was as high as 76.49% due to the hydrophobic effect and large surface area. More importantly, FA-BSA-PEG/MoOx nanosheets showed a pH-dependent oxidative degradation properties: the nanosheets are relatively stable at acidic pH and degradable at physiological pH, resulting in longer retention in tumor and minimal impact on normal tissues, thereby demonstrating a high level of safety to our body.

The antitumor results showed that the chemotherapy and PTT combined treatment using FA-BSA-PEG/MoOx could significantly increase the temperature of the primary tumor, induce ICD, and trigger a “switch” in tumor immune response. The TAAs and DAMPs, released by tumor cell death, enhance the immunogenicity of the tumor microenvironment, promote the dendritic cells (DCs) maturation, and alleviate the tumor immunosuppressive microenvironment. In addition, the nanosheet inhibited not only the primary tumor growth but also distant tumor growth (inhibition rate: 51.7%) and lung metastasis (inhibition rate: 93.6%), which is far more effective compared to the commercial Taxotere®. In sum, excellent results at the cell and animal levels suggest that this novel multifunctional degradable nanosheet is a promising platform to combine the PTT and chemotherapy for breast cancer treatment.

## Results and discussions

### The design strategy, fabrication and characterization of multifunctional nanosheet

**Scheme1:**
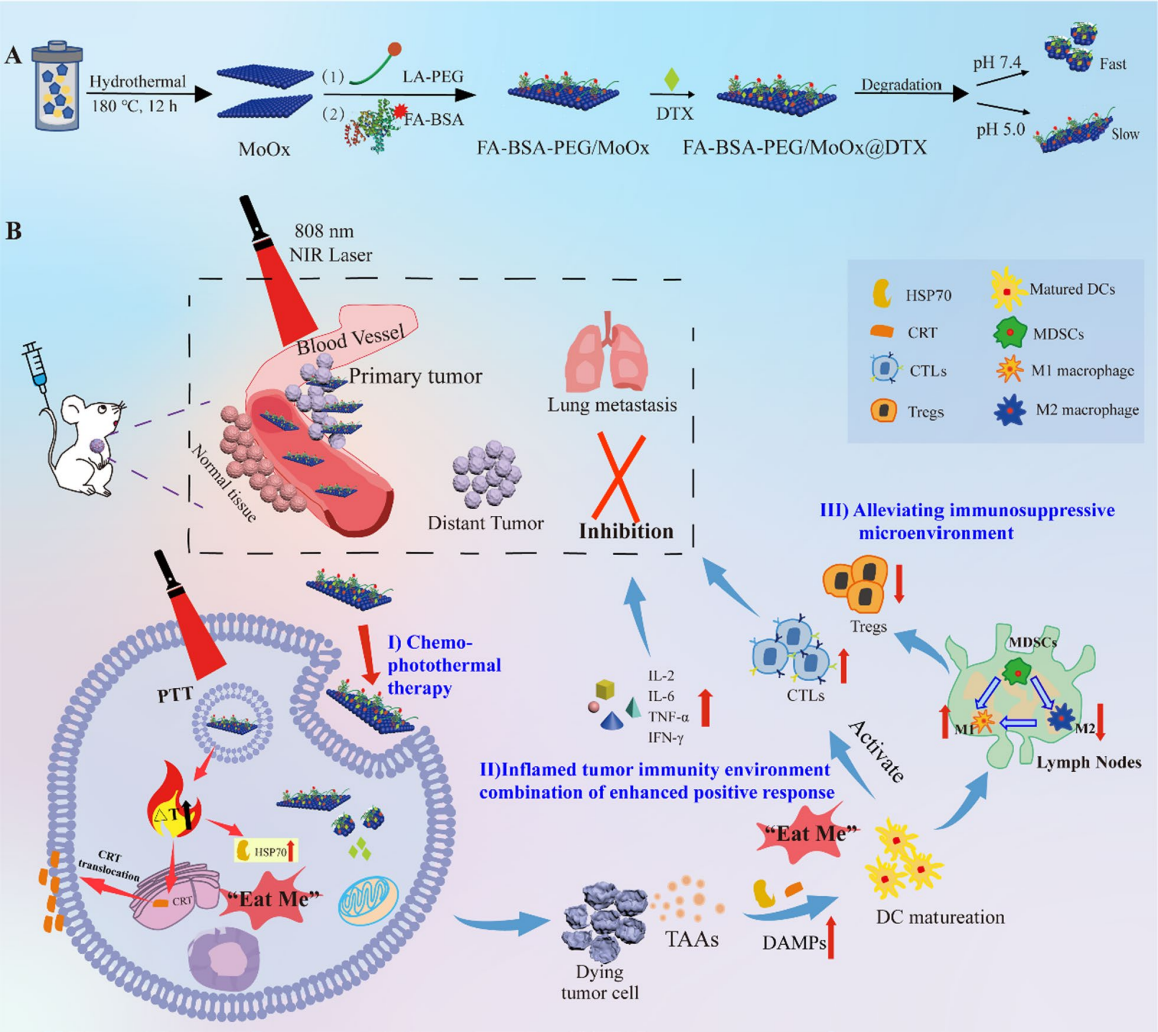
The schematic illustration of the multi-strategy for cancer treatment. **A** The preparation route of the FA-BSA-PEG/MoOx@DTX nanosheet and the in vitro antitumor and degradation experimental design; **B** the elucidation of the mechanism of FA-BSA-PEG/MoOx@DTX + NIR combination therapy for meliorating tumor immunosuppression, inhibiting distant tumor and lung metastasis

**Fig. 1:**
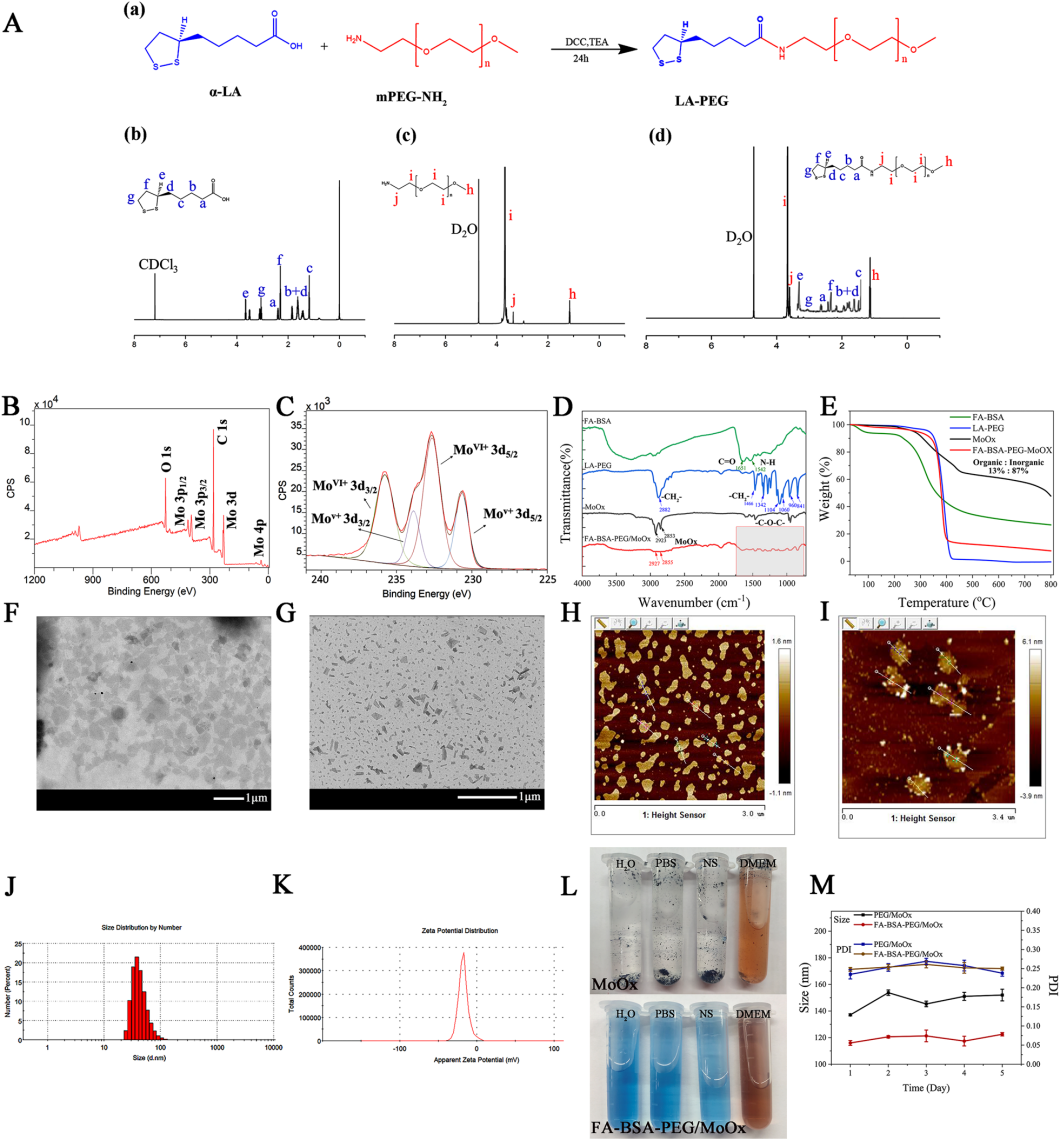
**A** The synthesis route of LA-PEG and ^1^H NMR spectra (α-LA, mPEG-NH_2_ and LA-PEG); XPS survey (**B**) and the high-resolution spectra of Mo 3d (**C**); **D** the TFIR spectra of FA-BSA, LA-PEG, MoOx and FA-BSA-PEG/MoOx; **E** thermogravimetric curves of FA-BSA, LA-PEG, MoOx and FA-BSA-PEG/MoOx; TEM and AFM images of MoOx (**F**, **H**) and FA-BSA-PEG/MoOx (**G**, **I**) nanosheets; particle size distribution (**J**) and Zeta potential (**K**) of FA-BSA- PEG/MoOx nanosheets; **L** the dispersion stability of MoOx and FA-BSA-PEG/MoOx nanosheets in different media (DI water, PBS, normal saline (NS), serum); **M** the long time stability of FA-BSA-PEG/MoOx nanosheets

The ultrathin blue molybdenum oxide nanosheets were synthesized by a hydrothermal method (Scheme 1A). The survey (Fig. 1B) and high resolution (Fig. 1C) spectra indicate the nanosheets consisted of both Mo^V^ and Mo^VI^, suggesting that the ammonium molybdate was partly reduced by the oleylamine in the autoclave forming the H_x_ (Mo^V^_x_)(Mo^VI^_1−x_) O_3_ (defined as MoOx). The typical four-peak-shaped Mo3d spectrum could be well fitted into two spin–orbit doublets: Mo^V^ 3d_5/2_ (230.76 eV), Mo^V^ 3d_3/2_ (234.06 eV), Mo ^VI^ 3d_5/2_ (232.85 eV) and Mo6 ^VI^ 3d_3/2_ (235.94 eV), and the ratios of Mo^V^ and Mo^VI^ was 69.53% and 30.47%, respectively, indicating that there are oxygen deficient area in the molybdenum oxide nanosheet. A dual-modified MoOx (FA-BSA-PEG/MoOx) nanosheet with excellent stability and targeting capability was then conducted for breast cancer combined with PTT and chemotherapy (Scheme 1B). In brief, the LA-PEG could conjugate to the MoOx surface by the interaction between thiol of LA-PEG and oxygen deficient area of the MoOx. The PEG could form a hydration layer with the water molecule and act as a shield preventing the aggregation; The FA-BSA, a solid stabilizer to improve biocompatibility, could anchor to the MoOx by the hydrophobic interaction, and expose the hydrophilic FA on the outer layer of the FA-BSA-PEG/MoOx nanosheet for the tumor targeting. The synergy of these effects was expected to achieve the goal of excellent anti-tumor and lung metastasis effect. More importantly, the pH-dependent degradability endowed the nanosheet with prolonged drug release time in the tumor and reduced accumulation in normal organs, offering great safety compared to the other inorganic materials.

The synthetic route and ^1^H-NMR spectrum of LA-PEG is shown in Fig. 1A and the assignment of hydrogen in LA-PEG is as follows: 2.46 (m, 2H, aH), 1.72, 1.61, 1.54, 1.38 (m, 2H, b + dH), 1.24 (d, 2H, cH), 3, 34 (s, 1H, eH), 2.13 (m, 2H, fH), 3.20 (m, 2H, gH), 1.14 (t, 3H, hH), 3.72 (m, 2H, iH), 3.53 (s, 2H, jH). For the FA-BSA, the grafting ratio of FA in FA-BSA is 10.32% (based on the Kaunas brilliant blue method, Additional file 1: Fig. S3).

The FTIR spectra of the different samples are shown in Fig. 1D and Additional file 1: Fig. S1. The wavenumber at 911 cm^−1^ of FA is attributed to the vibration of γ(–OH), which disappeared in the FA-BSA spectrum, and the new peaks at 1651 cm^−1^ ʋ (C=O), 1542 cm^−1^ ʋ (N–H) and 1045 cm^−1^ ʋ (C–N) also indicate the successful preparation of FA-BSA. The wavenumbers of 2882 cm^−1^, 1466 cm^−1^, and 1342 cm^−1^ are the characteristic absorption peaks of LA-PEG; 2923 cm^−1^ and 2853 cm^−1^ are the characteristic absorption peaks of MoOx, and the FA-BSA-PEG/MoOx spectrum contains all the above-mentioned characteristic peaks. Furthermore, the characteristic absorption peaks in the fingerprint area were red-shifted, as shown in Additional file 1: Fig. S2. The amounts of MoOx and the organic part in FA-BSA-PEG/MoOx were estimated to be about 13% and 87%, respectively using TGA (Fig. 1E). In summary, the above results confirmed that FA-BSA-PEG/MoOx nanosheets were successfully prepared. Furthermore, the spectrum shows no interference in the near-infrared region, indicating that the FA-BSA and LA-PEG are not weaken the photothermal conversion efficiency (Additional file 1: Fig. S4).

The MoOx and FA-BSA-PEG/MoOx nanosheets showed lamellar structures based on the morphology observations by TEM (Fig. 1F, G) and AFM (Fig. 1H, I). The diameter of the pristine MoOx was around 300 nm (estimated by TEM), while the diameter of the modified nanosheets was significantly reduced to 80.09 ± 1.45 nm (PDI = 0.265, Fig. 1J) with a zeta potential of − 17.5 ± 0.10 mv (Fig. 1K), which was confirmed by the TEM (Fig. 1G). The thicknesses of the pristine MoOx and FA-BSA-PEG/MoOx were 1 nm (Additional file 1: Fig. S5A) and 3 nm (Additional file 1: Fig. S5B), respectively. The increase in thickness was attributed to the wrap of FA-BSA and LA-PEG on the surface. The FA-BSA-PEG/MoOx could maintain stability in DI water, PBS, normal saline and serum medium (Fig. 1L), compared to pristine MoOx, which aggregated and formed a precipitate in four different media due to strong hydrophobic interactions. Moreover, FA-BSA-PEG/MoOx was highly stable in water for up to 5 days, and the size and polydispersity index (PDI) of nanoparticles were stable without significant changes (Fig. 1M), indicating its excellent stability, which is critical for in vivo applications.

### The photothermal performance and degradation properties in vitro

**Fig. 2:**
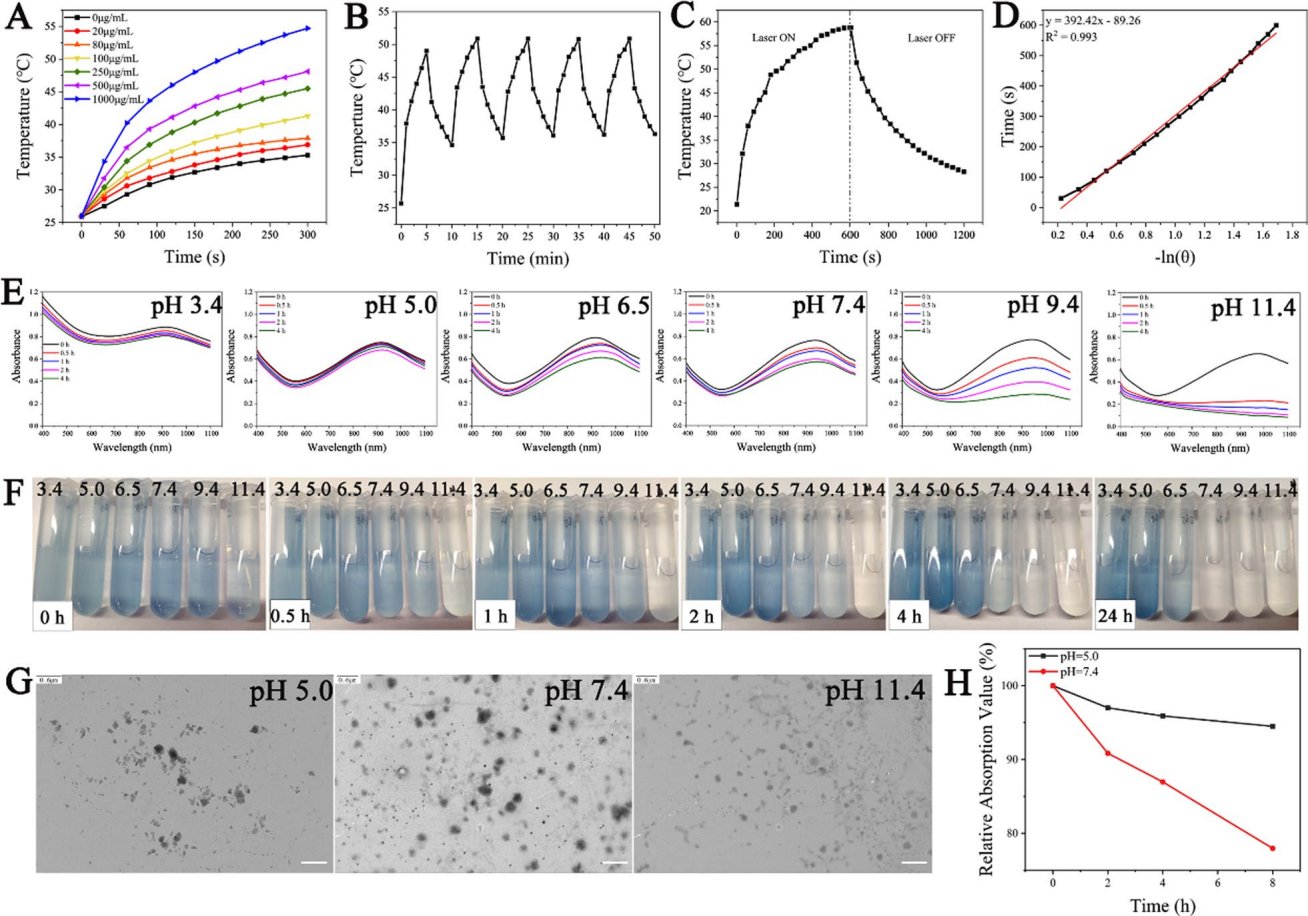
Photothermal and degradation properties of FA-BSA-PEG/MoOx nanosheets. **A** The temperature heating curves of nanosheets with different concentrations; **B** temperature variations of nanosheets solution under continuous laser irradiation for five on–off cycles; **C** temperature rise and fall curves of nanosheets with laser on/off; **D** photothermal conversion fitting curve of nanosheets; **E** degradation process of MoOx-PEG incubated in PBS with different pH values and **F** digital photos; **G** TEM images of nanosheets after 2 h degradation at pH 5.0, 7.4 and 11.4 PBS solution, respectively; scale bar: 0.6 μm; **H** the degradation curves of nanosheet in serum with different pH values (5.0, 7.4) over time

The FA-BSA-PEG/MoOx exhibited strong absorption from 700 to 1000 nm due to the localized surface plasmon resonance (LSPR) originating from the intervalence charge-transfer transition between Mo^V^ and Mo^VI^ [27], and the absorption showed high stability after being irradiated for different time periods (0, 10, 20 and 30 min) (Additional file 1: Fig. S6), which plays a vital role in cancer non-invasion treatment. The photothermal conversion capacity of the nanosheet displayed a significant concentration- and time-dependent behavior, and the highest temperature could be up to 55 °C (Fig. 2A and Additional file 1: Fig. S7), which is consistent with the reported data and facilitates synergistic cancer treatment [28, 29]. Furthermore, the FA-BSA-PEG/MoOx retained its excellent photothermal performance without any perceptible attenuation even after five cycles of exposure to NIR irradiation (Fig. 2B). After removal of the NIR treatment, the temperature decreased to normal (Fig. 2C). The photothermal conversion showed a linear correlation (Fig. 2D), and the FA-BSA-PEG/MoOx nanosheets showed a photothermal conversion rate of 43.41%. Taken together, the FA-BSA-PEG/MoOx nanosheets exhibited excellent photothermal conversion performance and are a promising candidate for cancer combined treatment.

For in vivo applications, biodegradability is an important factor in hampering the application of inorganic nanomaterials. The FA-BSA-PEG/MoOx nanosheet showed a pH-dependence (Fig. 2E, F and Additional file 1: Fig. S8,) in alkaline environments (pH = 9.4 and 11.4); the NIR adsorption showed a rapid decrease, revealing that FA-BSA-PEG/MoOx is unstable under alkaline conditions and degrades rapidly. This was attributed to the oxidation reaction of MoOx from H_x_ [(Mo^V^_x_)(Mo^VI^_1−x_) O_3_]^−^ to the [Mo^VI^O_4_]^2−^ state mediated by the generation of unstable intermediate product [(Mo^V^x)(Mo^VI^_1−x_) O_3_]^−^ in an alkaline environment. The final chemical species with the highest valence of MoO_4_^2−^ is in the ion state and could be filtrated by kidneys, which is critical to in vivo applications, and it could be completely metabolized without any toxicity to the body. The nanosheet had a lower degradation kinetics in neutral (pH = 7.4) and acidic conditions (pH = 3.4, 5.0, and 6.5) due to the lack of formation the unstable intermediate product [(Mo^V^x)(Mo^VI^_1−x_) O_3_]^−^. The same results could also be observed in the digital photographs, and the color showed the quickest fading trend in a pH 11.4 medium, which was attributed to the oxidation of Mo^V^ to Mo^VI^. The degradation properties were also confirmed by the TEM results, as shown in Fig. 2G, where the nanosheets are seen to remain relatively intact at pH 5.0 and pH 7.4; however, a lot of small fragments could be observed in the alkaline medium. Similar degradation behavior was observed in the serum medium (Fig. 2H, Additional file 1: Fig. S9A, B).

For drug loading, the nanosheet showed the maximum drug loading rate of 76.49% with an efficacy of 80% based on the HPCL assay (Additional file 1: Table S1) (the chromatogram of DTX is shown in Additional file 1: Fig. S10), which can be attributed to the larger surface area of the nanosheets and strong hydrophobic interactions between nanosheets and DTX. The sustained release properties in pH 5.0 medium could prolong the drug release time and increase the bioavailability of DTX (Additional file 1: Fig. S11, Table S4).

### Toxicity evaluation and cell uptake

**Fig. 3:**
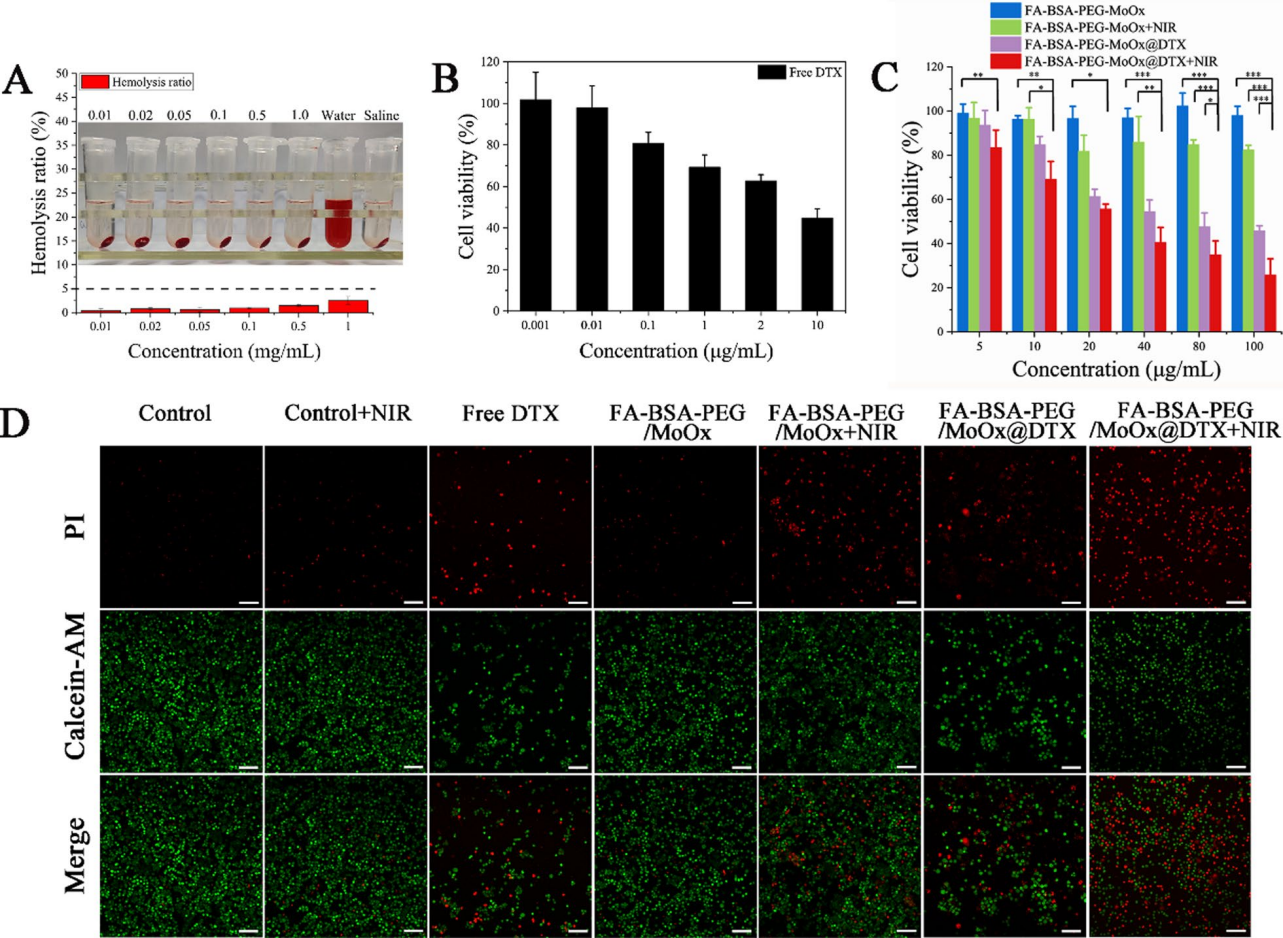
Toxicity of different agents/formulations. **A** The hemolysis and digital photos of HRBCs treated by FA-BSA-PEG/MoOx nanosheet with different concentrations. The cell viability of MCF-7 cells after treatment with different concentrations of formulations: **B** DTX; **C** FA-BSA-PEG/MoOx; FA-BSA-PEG/MoOx + NIR (808 nm, 1.5 W/cm^2^); FA-BSA-PEG/MoOx @DTX; FA-BSA-PEG/MoOx@DTX + NIR (808 nm, 1.5 W/cm^2^) (*p < 0.05, **p < 0.01, ***p < 0.001); **D** live and dead staining results after treatment with different formulations; the scale bar: 100 μm

As a novel intravenous injection drug delivery system, hemolysis is an essential indicator for the evaluation of its blood compatibility and is the first screen in a safety test. The hemolysis of FA-BSA-PEG/MoOx nanosheets with different concentrations was observed to be in the range of 0.40–2.54% (less than 5%), indicating that FA-BSA-PEG/MoOx nanosheets have good hemocompatibility (Fig. 3A, Additional file 1: Table S2).

Besides the hemolysis assay, the cytotoxicities of the FA-BSA-PEG/MoOx and DTX-loaded nanosheet with and without NIR radiation on MCF-7 cells were also evaluated by CCK-8 method. The cytotoxicity of free DTX exhibited a concentration-dependent manner, and the survival rate of cells decreases with the lowest value being 37% (Fig. 3B). The blank FA-BSA-PEG/MoOx nanosheet showed high viability (95%) even at a concentration up to 100 μg/mL, indicating an excellent cytocompatibility (Fig. 3C). The short time of NIR treatment appears to have a limited effect on killing cancer cells (80%) (Fig. 3D), and the FA-BSA-PEG/MoOx@DTX group showed a similar trend to the free DTX group (Fig. 3E). Combining chemotherapy and PTT treatments, an excellent in vitro anti-tumor ability with reduced cell viabilities compared to the same concentration of free DTX is also evidenced by the live/dead staining method as shown in Fig. 3F, G, indicating the promise of the synergistic effect of chemotherapy and hyperthermia.

**Fig. 4:**
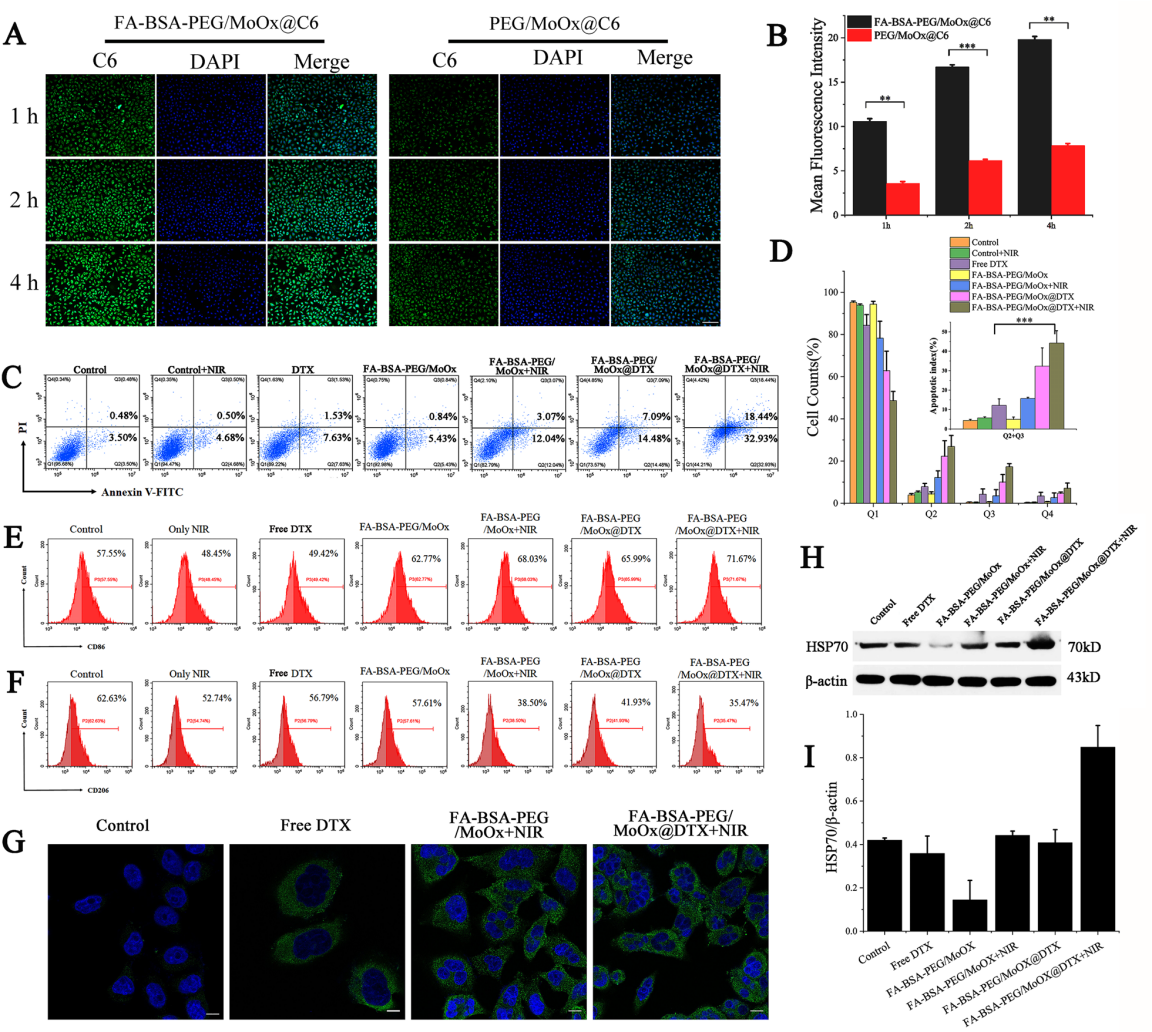
Representative fluorescent images (**A**) and a quantitative assay (**B**) of MCF-7 cells treated with FA-BSA-PEG/MoOx@C6, PEG/MoOx@C6 for 1, 2, 4 h; the scale is 100 μm; (*p < 0.05, **p < 0.01, ***p < 0.001). **C** Apoptosis results of MCF-7 cells treated with different preparations for 24 h; **D** quantitative analyses of apoptosis statistics results of MCF-7 cells treated with different formulations. The result of Q2 plus Q3 (n = 3); Flow cytometry analysis of the expression of **E** CD86 (M1 macrophage marker) and **F** CD206 (M2 macrophage marker) on macrophages treated by different formulations. **G** The CLSM images of CRT translocation after treated with different formulations. The green and blue represent CRT and nuclei, respectively. Scale bar: 10 μm; expression of HSP70 (**H**) and HSP70/β—actin ratio (**J**) after different treatments were compared

The tumor cell targeting ability of the FA-BSA-PEG/MoOx and PEG/MoOx nanosheets on MCF-7 cells was quantitatively studied by fluorescence microscopy and flow cytometry. The green fluorescence intensities (coumarin 6, C6) in the FA-BSA-PEG/MoOx group were remarkably stronger than that of PEG/MoOx-treated group (without FA modification) (Fig. 4A, B). These results demonstrate that the FA-BSA-PEG/MoOx@C6 could be effectively taken up by MCF-7 cells due to the high affinity of FA with the folate receptor on the cell surface. In addition, the fluorescence intensities increased with incubation time, indicating that the time-dependent uptake of the nanosheets by the cells, which is in agreement with the flow cytometry results (Additional file 1: Fig. S12).

### Cell apoptosis and immune assay

After uptake by the tumor cells, the ability to induce cell apoptosis was investigated using phosphatidylserine (PS) as the marker, which showed a membrane asymmetry state in the early stage of apoptosis. FITC labeled Annexin V was applied for apoptosis analysis. The value of Q2 (early apoptosis cells) plus Q3 (late apoptosis cells and necrotic cells) is defined as the apoptotic index, and the apoptosis index of the NIR treated group is 5.18%, which is similar to that of the control group (3.98%). The apoptotic index of the blank FA-BSA-PEG/MoOx nanosheets was 6.27%, which is in agreement with the results of cytotoxicity (Fig. 4C, D). In the FA-BSA-PEG/MoOx@DTX + NIR group, the apoptosis index increas to 51.37%, showing the great potential in inducing cell apoptosis combined with PTT and chemotherapy. Furthermore, after FA-BSA-PEG/MoOx@DTX + NIR treatment, M1 phenotype macrophages increased from 57.55 to 71.67%, and M2 macrophages decreased from 62.63 to 35.47% (Fig. 4E, F). These results are consistent with the reports in the literature [30]. The effective alleviation of the tumor immunosuppressive microenvironment is conducive to cancer treatment. During ICD, CRT, an “eat me” signal, is generally expressed in the endoplasmic reticulum and translocated to the cell surface, which stimulates the antigen presentation function of dendritic cells and activates a series of immune responses. The green fluorescence (CRT) in DTX group is significantly stronger than that of the control group, demonstrating that the DTX can induce ICD and lead to cell death, and these results are in accordance with the report in the literature [31, 32]. The FA-BSA-PEG/MoOx@DTX + NIR group combining the PTT and chemotherapy exhibited the strongest green fluorescent intensity compared to the free DTX and single NIR treatment group (Fig. 4G). These results demonstrate the promising future of combination therapy. However, the western blot results showed that the total amount of CRT protein remained basically unchanged after different sample treatments, with or without NIR treatment, as shown in Additional file 1: Fig. S13. The level of HSP 70 was examined via western blotting to assess the effect of different formulations on MCF-7 cells as shown in Fig. 4H, J. The combination treatment group significantly enhanced HSP70 expression, revealing that the NIR can not only kill the cancer cell via photothermal effect, but also release TAAs and DAMPs, triggering ICD.

### In vivo safety assessment and pharmacokinetic assay

**Fig. 5:**
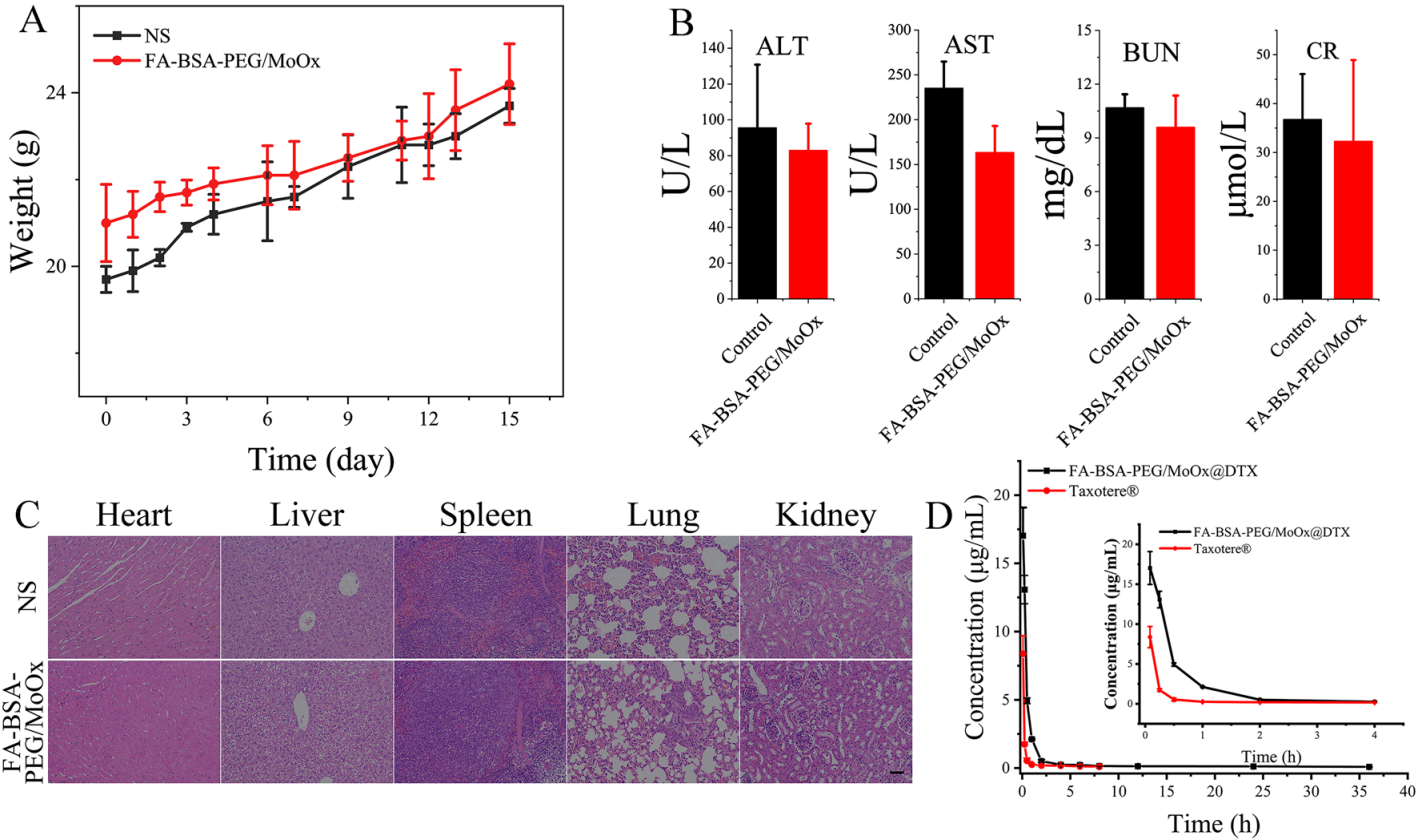
**A** Blood analysis of mice on day 15 post-injection of FA-BSA-PEG/MoOx nanosheets (20 mg/kg) or saline from the tail vein. Detection indicators: AST, ALT, BUN and CR; **B** the body weight change curve of mice after intravenous administration of FA-BSA-PEG/MoOx nanosheets (20 mg/kg) or saline for 15 days; **C** H&E stained images of main organs retrieved form mice treated by normal saline and FA-BSA-PEG/MoOx nanosheet; **D** plasma DTX concentration–time curve for intravenous administration of commercial Taxotere® and nanosheet in rats (n = 3)

Based on the hemolysis test in vitro, the hemocompatibility was further evaluated by whole blood analysis, and all inspection items were in the normal range (Additional file 1: Table S3), indicating an excellent hemocompatibility of the FA-BSA-PEG/MoOx nanosheet. Besides, the systemic toxicity was also evaluated by body weight and histological analysis. During the test period, all the animals showed a normal state and did not show any signs of distress or infection, and symptoms of toxicity like slow movement were not observed. The animals kept gaining weight over 15 days in both two groups (Fig. 5A). Additionally, the liver function indicators of aspartate aminotransferase (AST), alanine aminotransferase (ALT), and the kidney function indicators of blood urea nitrogen (BUN) and creatinine (CR) further verified that nanosheets have no hepatotoxicity or nephrotoxicity (Fig. 5B). Further, the H&E staining images of the main organs (heart, liver, spleen, lung, and kidney) showed no significant difference in the pathological analysis compared to the saline group (Fig. 5C), and symptoms of chronic injury were also not observed during the test period. In addition, four indicators, including ALT (Additional file 1: Fig. S14A), AST (Additional file 1: Fig. S14B), BUN (Additional file 1: Fig. S14C), and CR (Additional file 1: Fig. S14D) were selected to assess the toxicity of the drug-loaded nanosheet to the liver and kidney, and the values of the indicators in Taxotere® treated group was found to be significantly higher than those of the nanosheet group. Especially for the AST, the value is far beyond the upper limit of the normal range, indicating impaired liver function. These results demonstrate that the nanosheet can remarkably reduce the toxicity of the DTX as a result of the FA targeting properties.

Based on the above results, it can be concluded that the nanosheet has excellent compatibility, which is consistent with the in vitro results and shows great potential for biomedical applications.

Based on the excellent stability in vitro, in vivo stability was evaluated by the pharmacokinetic assay, with the commercial Taxotere® set as control. The pharmacokinetic results are shown in Table 1, Fig. 5D, and Additional file 1: Table S4. Compared to the commercial Taxotere®, the nanosheets significantly increase the maximum blood concentration (increased by 2.13 times) (Additional file 1: Table S4) and the area under the drug-time curve (AUC_0-t_) (increased by 4.54 times). In addition, the mean residence times (MRT, increased by 3.86 times) and half-times (increased by 4.14 times) were greatly extended, while the clearance rate was reduced to 23.3% of the Taxotere® group. These results are consistent with the previous in vitro results. It is well-known that the surface properties of the nanosheet is the critical factor in deciding its fate, and the synergistic effect of BSA and PEG on the surface of FA-BSA-PEG/MoOx can protect the nanosheet from being recognized and cleared by the macrophage, extending the circulation time in the bloodstream and increasing bioavailability.

**Table 1:** The main pharmacokinetic parameters of Taxotere® and FA-BSA-PEG/MoOx + DTX after intravenous administration

Parameters	Taxotere®	Nanosheet
AUC_(0-t)_ (mg/L*h)	3.29 ± 0.24	10.68 ± 0.58***
AUC_(0-∞)_ (mg/L*h)	4.13 ± 0.06	15.28 ± 1.66**
MRT_(0-t)_ (h)	1.46 ± 0.14	7.38 ± 0.32***
MRT_(0-∞)_ (h)	4.65 ± 0.83	31.01 ± 6.71*
t_1/2_z (h)	5.72 ± 0.78	35.06 ± 7.50*
CLz (L/h/kg)	2.42 ± 0.04	0.66 ± 0.08***
C_max_ (mg/L)	8.37 ± 1.33	17.62 ± 2.06**

Asterisks (*) denote statistical significance of nanosheet vs. commercial Taxotere® (*p < 0.05, **p < 0.01, ***p < 0.001)

### Biodistribution and photothermal effect

**Fig. 6:**
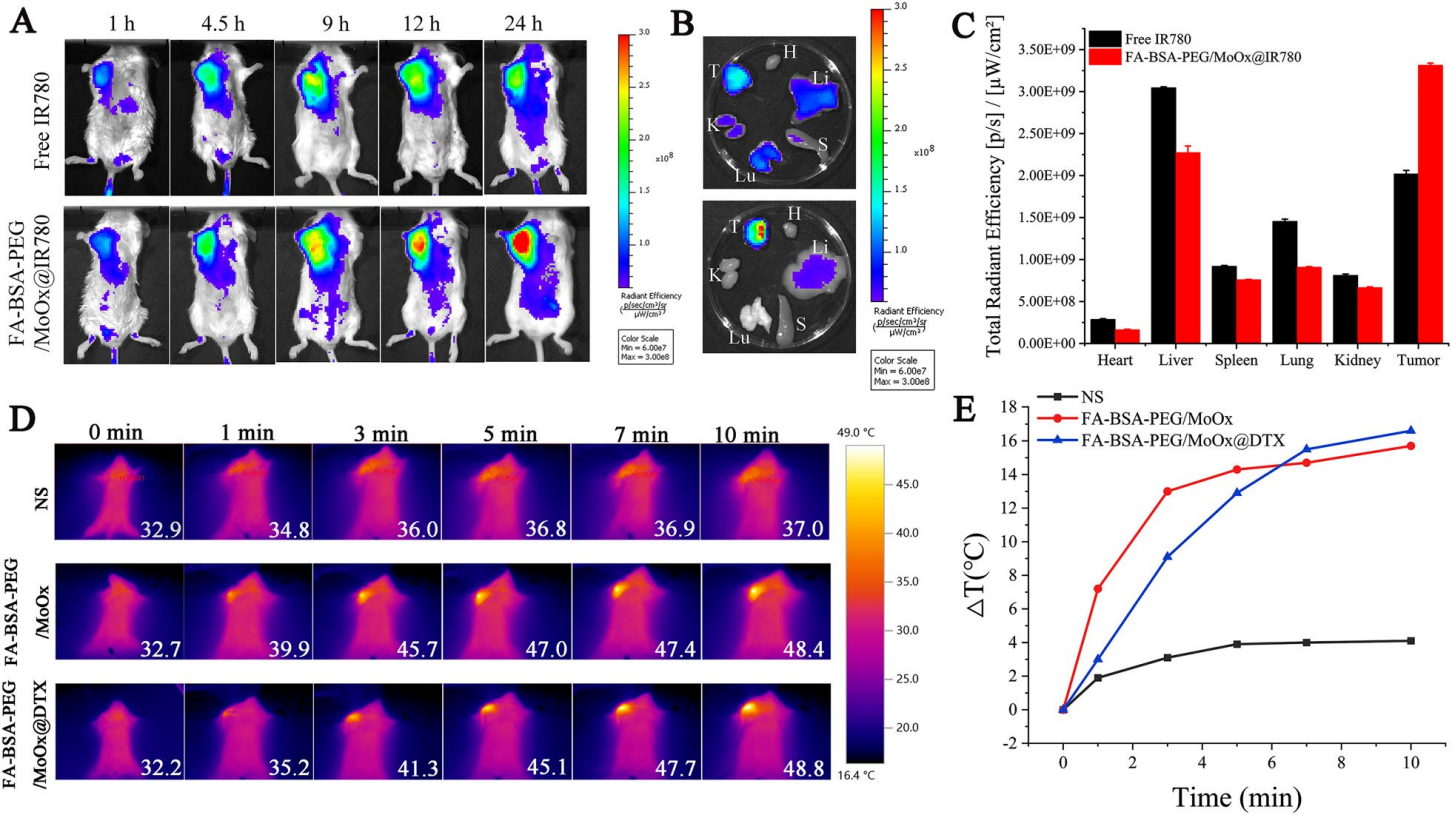
In vivo biodistribution and tumor targeting effect of nanosheet. **A** FMI images of tumor-bearing mice after intravenous administration of IR780 and FA-BSA-PEG/MoOx@IR780 at different time points; **B** ex vivo FMI images of the main organs retrieved from tumor-bearing mice at 24 h time point after intravenous administration of IR780 and FA-BSA-PEG/MoOx@IR780. The H, Li, S, Lu, K, and T represent the heart, liver, spleen, lung, kidney, and tumor, respectively; **C** quantitative analysis results of ex vivo mean fluorescence intensity of tumors and main organs. The near-infrared thermal images of the mice (**D**) and the temperature changes of the tumors (**E**) by intravenous administration of normal saline, FA-BSA-PEG/MoOx and FA-BSA-PEG/MoOx@DTX, respectively

The IR780 was selected as the fluorescence molecular imaging (FMI) for the biodistribution assay, and IVIS Kinetic small-animal imaging system was used to investigate the tissue distribution of free IR780 and FA-BSA-PEG/MoOx@IR780 after tail vein administration (Fig. 6A). In the free IR780 group, the fluorescence intensity reached the peak at 9 h post-administration, and then spread to the body gradually. In contrast, the fluorescence intensity of FA-BSA-PEG/MoOx@IR780 at the tumor site gradually increased, reaching the maximum at 24 h, which showed significantly extended circulation time, and this result was consistent with the pharmacokinetic test. Also, the fluorescence intensity of FA-BSA-PEG/MoOx@IR780 nanosheets at the tumor site was significantly stronger than that of free IR780 at all the time points, indicating that the FA-BSA-PEG/MoOx nanosheets could be accumulated in the tumor site effectively due to the FA targeting effect and the increased circulation time (Fig. 6B, C).

Based on the in vivo distribution results, the mice were treated by NIR at 24 h post-administration. The mice of the intra-tumor administration group were exposed to the NIR immediately after administration. The NIR thermal images are shown in Fig. 6D, and the heating curve was shown in Fig. 6E. After 10 min of continuous irradiation, the tumor temperature in the saline group only increased by 4.1 °C. However, in the FA-BSA-PEG/MoOx and FA-BSA-PEG/MoOx@DTX groups, the tumor temperature can rise rapidly up to above 42 °C only after 3 min’ irradiation and then reach the maximum temperature of 48.4 °C and 48.8 °C, respectively, demonstrating the excellent photothermal properties of the nanosheets in vivo.

As an alternative administration method, the nanosheet was also injected into the tumor directly. In this case, the temperature of the FA-BSA-PEG/MoOx and FA-BSA-PEG/MoOx@DTX groups rises up to 55.3 °C and 57.5 °C, respectively, showing a higher temperature compared to that of the vein administration group (Additional file 1: Fig. S15A, B). This can be attributed to the high concentration of the nanosheet without any loss in the blood circulation. The high temperature of the tumor site is conducive to enhancing the blood flow inside the tumor and increasing the permeability of the cell membrane, which can facilitate the internalization of the drug and deeper penetration into the deeper of the tumor tissue [27]. Both of the two administration methods verified that the nanosheet has a great photothermal conversion potential in vivo.

### Anti-tumor effect and lung metastasis in vivo

**Fig. 7:**
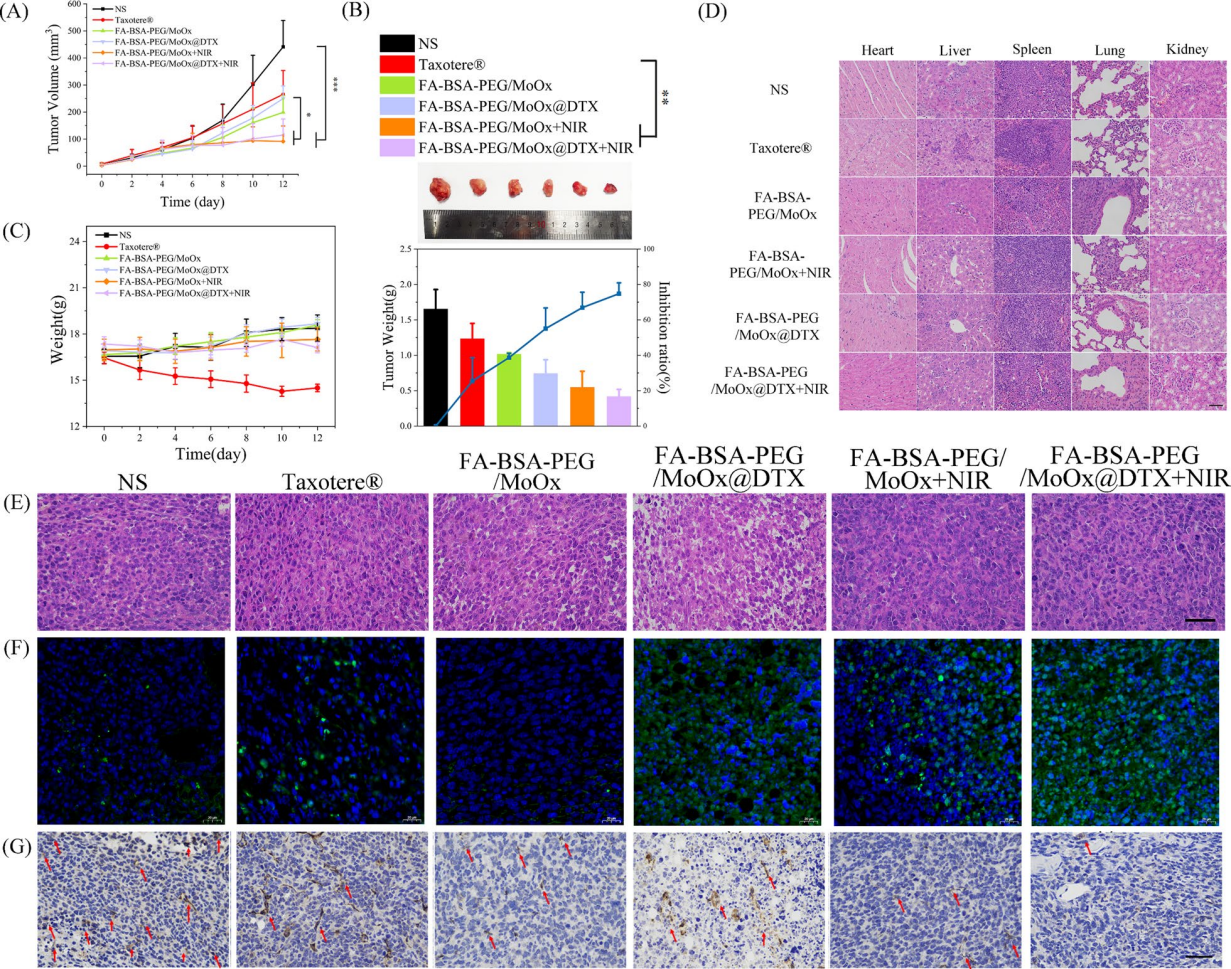
The in vivo photothermal therapeutic effect. **A** Mean tumor volume change curve after different treatments; **B** tumor weights and images, and tumor inhibition rate of mice in different treatment groups on day 12; **C** body weight change curve in different treatment groups; scale bar: 200 μm; **D** the H&E staining images of the main organs treated by various groups; The tumor tissue analyzed by H&E staining (**E**) (scale bar: 200 μm) and TUNEL staining (**F**) (scale bar: 20 μm), respectively; **G** CD31 immunohistochemical staining of tumor tissue in different treatment groups. The red arrow points to the blood vessels; the scale bar: 200 μm

A unilateral 4T1 tumor-bearing BALB/c mice model was established; after treatment with different agents/formulations, the tumor volume of the mice was observed to be in a sequence of normal saline > Taxotere® > FA-BSA-PEG/MoOx > FA-BSA-PEG/MoOx@DTX > FA-BSA-PEG/MoOx + NIR > The FA-BSA-PEG/MoOx@DTX + NIR (Fig. 7A and Additional file 1: Fig. S16). Unexpectedly, the commercial Taxotere® treated group showed a relatively larger tumor and weight loss, which might be caused by the rapid clearance in the bloodstream and severe side effects due to the non-selective properties (inhibition rate 25.41%). In contrast, the FA-BSA-PEG/MoOx@DTX + NIR group showed the best antitumor effect, with an inhibition rate of 74.78%, and relatively stable body weight, implying that the nanosheets can reduce the toxicity and side effects (Fig. 7B, C).

In addition, the H&E staining images of the main organs show that there is no significant difference in the nanosheet group compared with the control group, indicating that the nanosheets have excellent biocompatibility and biosafety (Fig. 7D). However, in the PTT and chemotherapy-treated tumors, there are obvious cell membrane or nucleus changes and fibrosis, indicating that the DTX-loaded nanosheets can cause tumor cell death (Fig. 7E). The strongest green fluorescence and the largest amount of apoptosis bodies were observed in TUNEL images (Fig. 7F). In tumor tissue, differentiated and undifferentiated new blood vessels can be observed. Immunohistochemistry was also applied for the evaluation of the angiogenesis in tumor tissue, and CD31, a platelet endothelial cell adhesion factor and vascular marker, was selected to evaluate the endothelial cell movement and vascular formation. The normal saline group showed more CD31 expression, indicating the rapid growth of the tumor. The FA-BSA-PEG/MoOx@DTX + NIR group showed the least CD31 expression (Fig. 7G), which further confirmed that the combined PTT and chemotherapy effectively inhibit tumor growth by promoting tumor cell apoptosis and cell death.

**Fig. 8:**
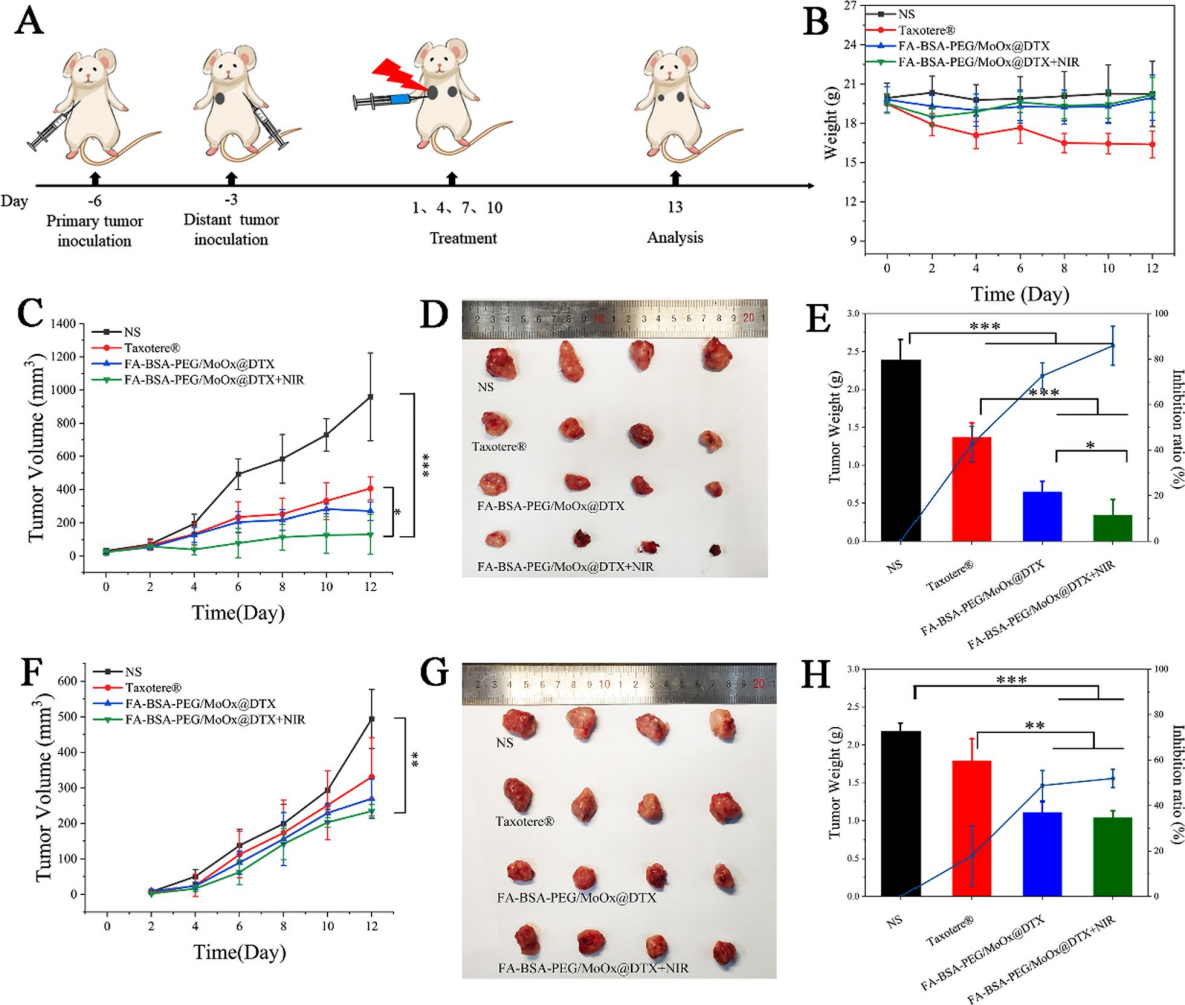
The in vivo antitumor (primary and distant tumors) evaluation. **A** A schematic illustration of the experimental design strategy; **B** body weights of 4T1-tumor bearing living mice after various treatments. The volume (**C**) and photos (**D**) of the primary tumor; **E** the weight and inhibit ratio of the primary tumor; The volume (**F**) and photos (**G**) of the distant tumor; **H** the weight and inhibit ratio of the distant tumor

The potential inhibition on the distant tumor was studied in a dual-armpit tumors model with two tumors in the left (the primary tumor) and right armpit (distant tumor) on G57BL mice. The experimental design and the treatment methods are shown in Fig. 8A, and the weight changes of the mice during the treatment period were consistent with the results of the above primary tumor inhibit study (Fig. 8B). The Taxotere®treated mice showed weight loss, and the weight of the mice in the other group showed no significant changes. The tumor volume results demonstrated that the primary tumor can be effectively inhibited (Fig. 8C–E); and distant tumor volume was relatively bigger than the primary tumor (Fig. 8F–H). The distant tumor inhibition rates of Taxotere® group, FA-BSA-PEG/MoOx@DTX and FA-BSA-PEG/MoOx@DTX + NIR were 17.85%, 48.80% and 51.92%, respectively.

**Fig. 9:**
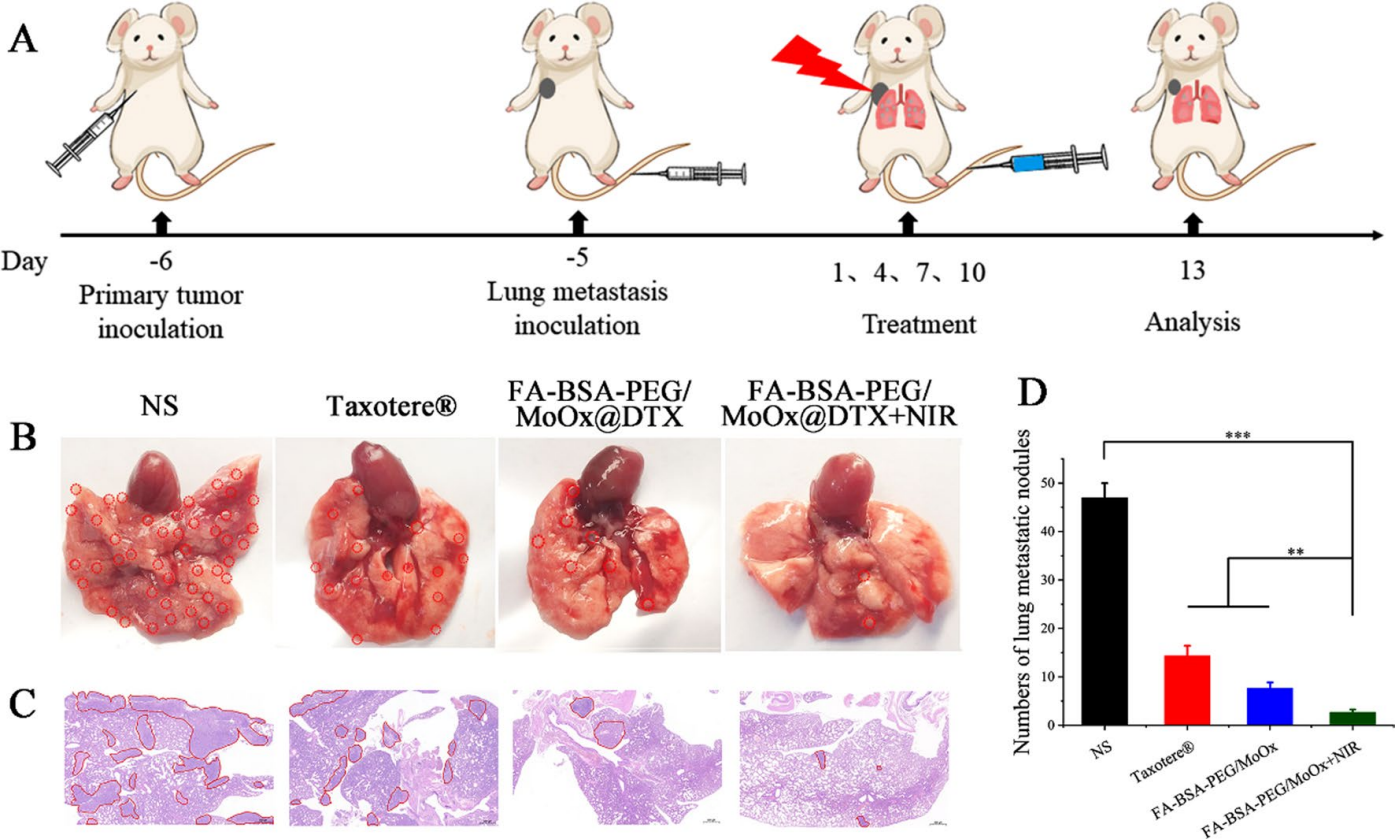
The in vivo lung metastases evaluation. **A** A schematic illustration of the experimental design strategy. The digital photos (**B**) and H&E images (**C**) of lung tissue in various treatment groups; the red circles represent lung nodules. Scale bar: 500 μm; **D** the lung nodule number in each groups

A breast cancer-based lung metastasis model was also constructed to evaluate the inhibitory effect of nanosheets on breast cancer lung metastasis. The experimental design and methods are shown in Fig. 9A. After treatment with different agents/formulations, there were obvious lesions and bulges in the normal saline and Taxotere®-treated group (Fig. 9B), in which the red circle represents lung nodules, indicating that free DTX had little effect on inhibiting tumor metastasis. However, the number of lung nodules in the FA-BSA-PEG/MoOx@DTX + NIR treated mice is significantly reduced (Fig. 9D). The H&E stained images further confirm similar results as shown in Fig. 9C, where the red circle represents the lung nodules, verifying that the combined therapy can suppress cancer cell metastasis. The other main organs and tumor tissue were also retrieved and stained by the H&E, as shown in Additional file 1: Fig. S17. The FA-BSA-PEG/MoOx@DTX + NIR group shows negligible damages to the liver, kidney and spleen tissues compared to the DTX groups. However, in the tumor tissue, the combination therapy group exhibited a significantly stronger inhibitory effect in the tumor tissue compared to the other three groups.

### The molecular mechanism from the perspective of immune response

**Fig. 10:**
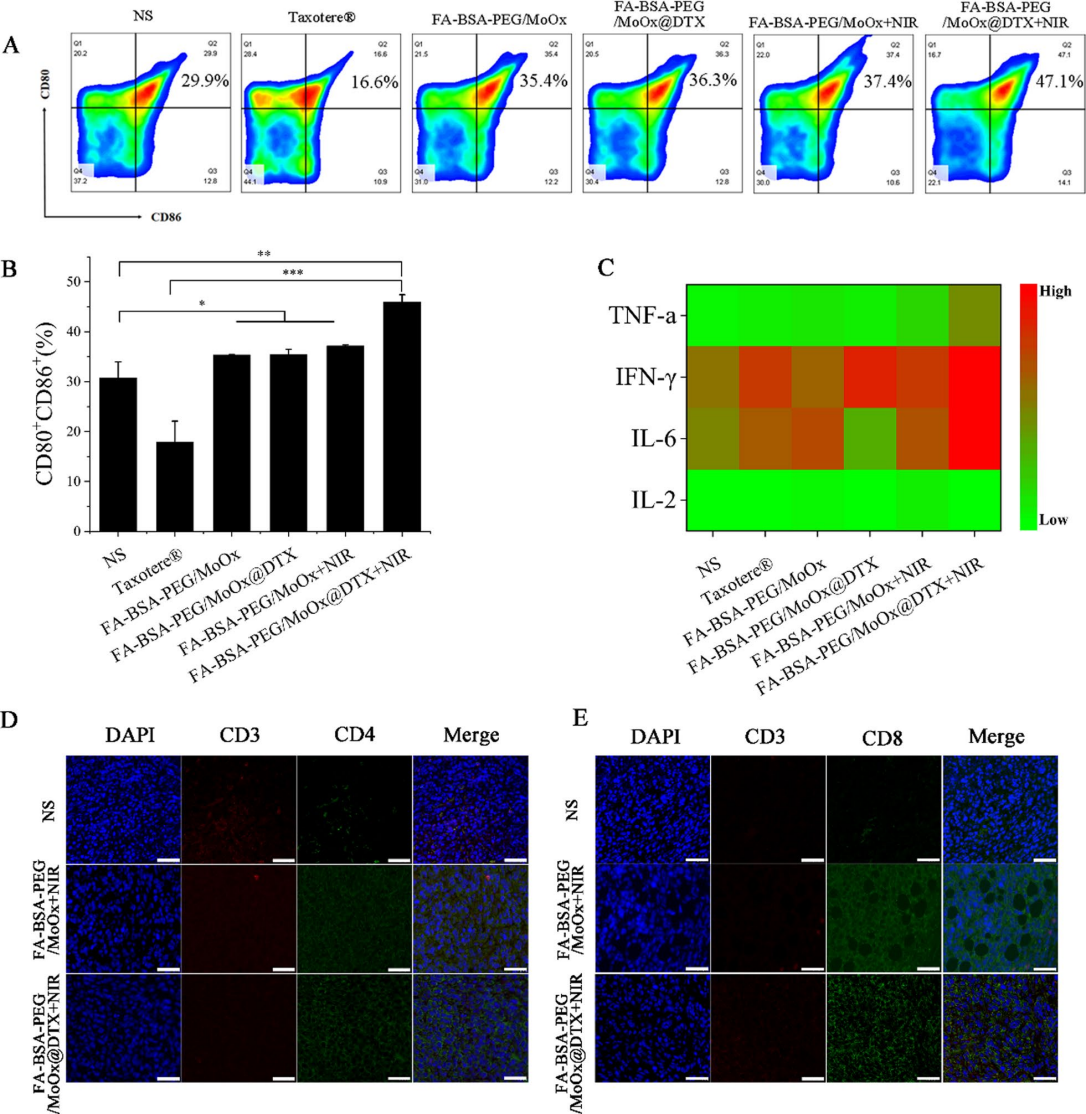
The mechanism of immune-promoting effects of nanosheet based on combined PTT and chemotherapy in a primary tumor model. Flow cytometry images (**A**) and quantitative analysis (**B**) of mature DCs (CD80^+^CD86^+^) in tumor-draining lymph nodes; **C** serum cytokine levels in the serum of tumor-bearing mice treated by different formulations; the CD4^+^ (**D**) and CD8^+^ CTLs (**E**) analysis of mouse tumor by immunofluorescence staining. Scale bar: 20 μm

**Fig. 11:**
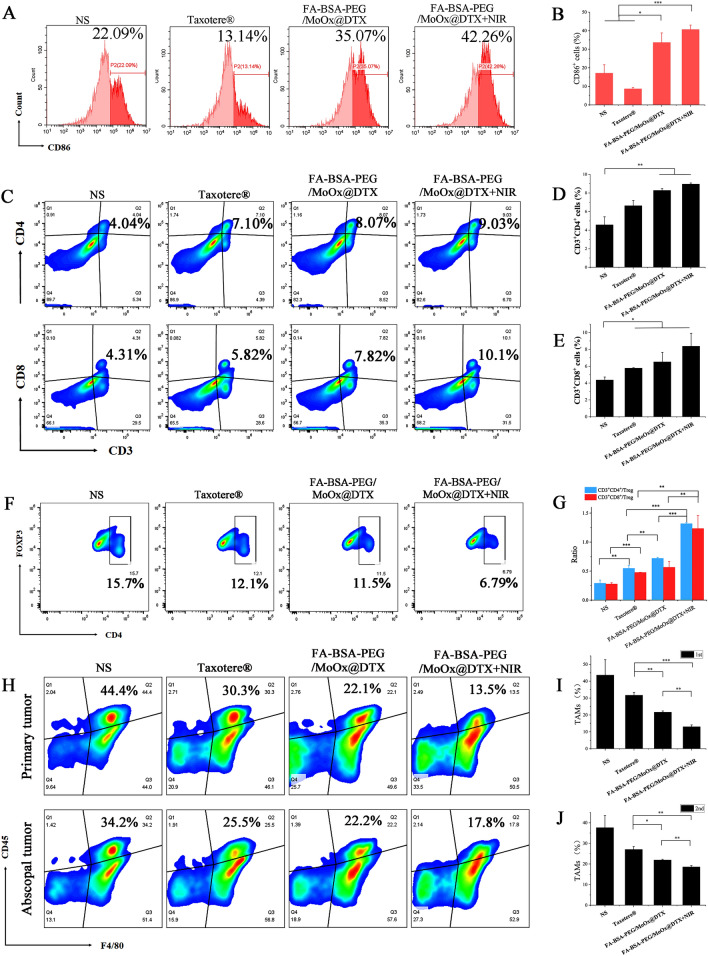
The mechanism of distant antitumor effect analysis. The flow cytometry images of DCs (CD80^+^CD86^+^) mature (**A**) and quantitative analysis (**B**); flow cytometry (**C**) and quantitative analysis (**D**, **E**) of activated CD3^+^CD4^+^CD8^+^ T cells after each treatment; the population of CD4^+^Foxp3^+^ Tregs in the spleen according to flow cytometry (**F**) and the ratio of CD3^+^CD8^+^ cytotoxic T cells versus CD3^+^CD4^+^Foxp3^+^ Tregs (**G**); flow cytometry analysis of CD45^+^, F4/80^+^ (**H**) and quantitative analysis (**I**, **J**) in the distant tumor

To understand the inhibition of the treated primary tumor, untreated distant tumor and lung metastasis, the molecular mechanism from the perspective of immune response was studied. As the antigen-presenting cells, the DCs cell has a unique function of inducing primary immune responses in the body, and the DCs from mouse inguinal drainage lymph nodes (LNs) were extracted for DCs mature analysis (Fig. 10A). There is an increase in the frequency of CD11c^+^CD80^+^ and CD11c^+^CD86^+^ cells in the DCs from mice LNs of the FA-BSA-PEG/MoOx@DTX + NIR combination therapy group, compared with that in the control group (increased by ~ 2.83, ~ 1.29 and ~ 1.25 times relative to the mature DCs in the commercial Taxotere®, FA-BSA-PEG/MoOx@DTX, FA-BSA-PEG/MoOx + NIR groups) (Fig. 10B). Meanwhile, as an important indicator in tumor immunity, the M1 type cytokines, including TNF-α, IFN-γ, IL-2 and IL-6, which promotes the killing effect of CTLs, were significantly higher in the FA-BSA-PEG/MoOx@DTX + NIR treated groups than those in the other three groups (Fig. 10C). In particular, the IFN-γ and IL-6 values are significantly up-regulated, indicating the inflamed state and meliorated tumor immunosuppression in the tumor. These results showed the inflamed tumor immunity environment, facilitating the killing of tumor cells. Furthermore, the immunofluorescence staining results of CD3^+^CD4^+^ helper T cells (Fig. 10D) and CD3^+^CD8^+^ cytotoxic T cells (Fig. 10E) show that the strongest green fluorescence intensity (More CD3^+^CD4^+^ helper T cells and CD3^+^CD8^+^ cytotoxic T cells) was in the FA-BSA-PEG/MoOx@DTX + NIR treated group, which indicates that more responder was generated to trigger the immune response and enhance antitumor immunity, leading to synergistically strengthened antitumor immunity. Moreover, the nanosheets were found to have an inhibition effect on the untreated distant tumor (51.7%) and on lung metastasis (93.6%). Therefore, we hypothesize that the FA-BSA-PEG/MoOx@DTX + NIR nanosheet can stimulate systemic antitumor immunity. In the bilateral 4T1 tumor-bearing mouse model, a similar result trend was observed: the percentage of the mature DCs in the chemotherapy and PTT combined treatment was 42.26%, which is 3.2 and 1.5 times values for the free DTX and FA-BSA-PEG/MoOx@DTX groups (Fig. 11A, B). An increase in the population of CD3^+^CD4^+^ helper T cells and CD3^+^CD8^+^ cytotoxic T cells was observed (Fig. 11C–E). In addition, the population of CD3^+^CD4^+^Foxp3^+^ Treg dramatically decreased to 6.79% compared to the commercial Taxotere® value of 12.1% (Fig. 11F). The ratio of CD3^+^CD8^+^ cytotoxic T cells to CD3^+^CD4^+^Foxp3^+^ Tregs was also found to be significantly increased in the combination-treated group (Fig. 11G). These findings indicate that BSA-PEG/MoOx@DTX + NIR can increase the CD3^+^CD8^+^ cytotoxic T cells and decrease the CD3^+^CD4^+^Foxp3^+^ Tregs, resulting in enhanced antitumor immunity. Furthermore, the percentage of CD45^+^F4/80^+^ cells in the chemotherapy and PTT combined treatment group is significantly reduced in both the primary tumor and the distant tumor compared with the Taxotere® group (Fig. 11H–J), indicating the relieved immunosuppression and enhanced immune response.

Combining the in vitro (Macrophage polarization, CRT and HSP 70 assays) and in vivo (DCs mature, T cell infiltration, cytokine assays) analysis results, it can be concluded that the hyperthermal tumor damage caused by the MoOx would be conducive for the deeper penetration of DTX tumor, which is an excellent synergistic effect for killing tumor cells. Furthermore, the FA-BSA-PEG/MoOx@DTX + NIR combination treatment could up-regulate the expression of DAMPs, TAAs, leading to DCs mature and amplifying ICD. This inflamed tumor immunity environment in combination with enhanced positive response, weakened immunosuppression, and enhanced systemic anti-tumor immunity, contributes to the extraordinary tumor inhibition effect on the treated primary tumor and well control of the untreated distant tumor and lung metastasis.

## Conclusion

In summary, a multifunctional chemotherapy and PTT platform, based on PEG and BSA decorated MoOx, was developed as a novel drug delivery carrier. The dual modified FA-BSA-PEG/MoOx nanosheet showed extraordinary stability in serum, which plays a paramount role for in vivo application, as proven by the biodistribution and pharmacokinetic tests. Upon reaching the tumor area, the FA could target the tumor cell by the specific receptor on the cell membrane. FA-BSA-PEG/MoOx, as an effective NIR photothermal agent, then significantly increase the temperature at the tumor site, allowing chemotherapy and PTT to work synergistically. The anti-tumor assay demonstrated that the FA-BSA-PEG/MoOx@DTX nanosheets combined photothermal chemotherapy could not only inhibit the primary tumor growth but also suppress distant tumor growth (inhibition rate: 51.7%) and lung metastasis (inhibition rate: 93.6%), demonstrating a substantially increased effectiveness compared to the commercial Taxotere®. Exploration of the molecular mechanism showed that in vivo immune response induced an increase in positive immune responders, suppressed negative immune suppressors, and established an inflammatory tumor immune environment, which contributes towards effectively suppressing tumor and lung metastasis. Most importantly, the pH-dependent degradability lead to longer retention time in tumors while ensuring minimal impact on normal tissues, demonstrating a high degree of safety. In conclusion, this novel multifunctional nanosheet has substantial promise as a platform for combination with chemotherapy and PTT for breast cancer treatment.

## Materials and methods

### Materials

Hydrochloric acid, cyclohexane, ethanol, triethylamine, tween-80, Methyl tert-butyl ether were purchased from China National Medicines Corporation Ltd; Dimethyl sulfoxide (DMSO), dichloromethane, methanol were purchased from Tianjin Fuyu Fine Chemical Co., Ltd; Ammonium Molybdate Tetrahydrate, Oleylamine, folic acid, NHS, EDC·HCl, α-lipoic acid were purchased from Shanghai aladdin reagent Co., Ltd; Docetaxel (114977-28-5) was purchased from Tianjin Xiensi biochemical technology Co., Ltd. Bovine serum albumin was purchased from Klon tech; mPEG-NH_2_ (MW5000) was purchased from Shanghai seebio biotechnology Co., Ltd; All reagents were used without further purification. 100× Penicillin–Streptomycin–Gentamicin Solution (P1410), PBS, Trypsin–EDTA solution, 0.25% (without phenol red) (9002-07-7), Calcein AM/PI live/dead cell double staining kit (CA1630), 5% BSA Blocking Buffer (SW3015), Percoll Cell separation solution (65455-52-9), Red blood cell lysis buffer (R1010), Collagenase IV (9001-12-1), DNase I (9003-98-9) were purchased from Beijing Solarbio Science & Technology Co., Ltd; Cell counting kit-8 (Catalog No. K1018) was purchased from APExBIO technology Ltd.; DAPI staining solution (C1002) was purchased from Beyotime biotechnology; Annexin V-FITC/PI apoptosis detection kit (40302ES50) was purchased from Shanghai Yisheng biotechnology Co., Ltd.; Fetal bovine serum (11011-8611) was purchased from Zhejiang tianhang biology Co., Ltd; Dulbecco's modified eagle medium (30030), Roswell Park Memorial Institute (RPMI) 1640 medium, CD11b monoclonal antibody (M1/70), APC (17-0112-82), FOXP3 monoclonal antibody (3G3), alexa fluor 647 (MA5-18160), CD11c monoclonal antibody (N418), FITC (11-0114-85), CD80 (B7-1) monoclonal antibody (16-10A1), APC (17-0801-82), CD86 (B7-2) monoclonal antibody (GL1), PE (12-0862-82), CD3 monoclonal antibody (17A2), APC (17-0032-80), CD8a monoclonal antibody (53–6.7), PE (12-0081-85), CD4 monoclonal antibody (GK1.5), FITC (11-0041-81), CD45 monoclonal antibody (30-F11), FITC (11-0451-82), F4/80 monoclonal antibody (BM8), APC (17-4801-80) were purchased from eBioscienceTM; APC anti-mouse CD86 (105011), PE anti-mouse CD206 (MMR) (141705), Recombinant mouse IL-4 (carrier-free) (574302) were purchased from BioLegend; Calreticulin polyclonal antibody (27298-1-AP) was purchased from Proteintech; TNF-α, IFN-γ, IL-6, IL-2 ELISA kits (EK202/2-48) were purchased from Multisciences (Lianke) Biotech, Co., Ltd.

### Cell lines

Human breast cancer cells (MCF‐7) and Mouse breast cancer cells (4T1) were obtained from Shanghai Zhong Qiao Xin Zhou Biotechnology Co., Ltd. 4T1 and MCF‐7 cells were incubated in RPMI‐1640 and DMEM media, respectively, supplemented with streptomycin and penicillin (1%). Both culture media were supplemented with 10% (v/v) fetal bovine serum (FBS). All cells were cultured in a 37 °C incubator with 5% CO_2_.

### Animals

Female BALB/c mice (aged: 6–8 weeks) were supplied by SPF (Beijing) Biotechnology Co., Ltd. All experiments were implemented according to the Animal Management Rules of the Ministry of Health of the People’s Republic of China and the Animal Experiment Ethics Review of Shandong University.

### Methods

#### Preparation of the nanosheets

##### Preparation of the MoOx

The MoO_X_ nanosheet was synthesized according to the literature [33]. Briefly, 1.23 g of ammonium molybdate was dissolved in 13 mL of deionized water (DI water), and then 1.2 mL of hydrochloric acid, 0.8 g of oleylamine (dissolved in 4 mL dichloromethane) was added to form a white emulsion, which was transferred into an autoclave and reacted at 180 °C for 12 h. The crude product was purified by ethanol and dried under a vacuum oven.

##### Synthesis of the folic acid (FA) modified BSA (FA-BSA) and the α-lipoic acid (LA) modified PEG (LA-PEG)

Both the FA-BSA and LA-PEG were synthesized based on the amidation reaction. FA-BSA: 200 mg FA, 434.3 mg EDC·HCl, and 260.7 mg NHS were dissolved in anhydrous DMSO, and then, the pre-activated FA-NHS was added dropwise to 10 mL of BSA solution and reacted for 24 h, and the final system was transferred into dialysis bag and dialyzed in 0.1 M PBS for 3 days and DI water for 3 days. The dialysis solution was lyophilized to obtain the FA-BSA complex, and the FA grafting ratio was calculated by UV–Vis spectrophotometer [34, 35]. LA-PEG: 90 mg LA and 1.0 g mPEG-NH_2_ were dissolved in 4 mL dichloromethane containing 20 mg DCC and 12 μL triethylamine (TEA) and reacted for 24 h. The mixture was then dried by the nitrogen blow, and the crude product was dissolved in DI water and extracted by dichloromethane. The purified product was lyophilized to obtain the solid LA-PEG [36].

##### Preparation of FA-BSA-PEG/MoOx

10 mg of MoOx nanosheets was accurately weighed and dispersed in 10 mL of cyclohexane. Then the system was sonicated (400 W, 2 s/2 s) for 60 min to obtain the uniform nanosheet. Subsequently, LA-PEG and FA-BSA were added and sonicated with the same condition to obtain the FA-BSA-PEG/MoOx.

#### Physicochemical properties and cell evaluation

##### Characterization

The ^1^H-NMR spectra of LA-PEG and FA-BSA were recorded on a 400 MHz spectrometer (AVANCE III, Germany) using deuterated dimethyl sulfoxide (DMSO-d6) or D2O as solvent. The Fourier transform infrared (FT-IR) spectra of LA-PEG and FA-BSA were obtained using an IR spectrometer (Nicolet iN10, America) with KBr as the substrate. The thermogravimetric analysis (TGA) (TGA/DSC1/1100LF, Switzerland) spectra of LA-PEG and FA-BSA were performed with the range of 20 °C to 800 °C, and a heating rate of 10 °C/min. An ultraviolet–visible spectrophotometer (UV-8000, China) was applied to determine adsorption wavelength and to calculate the grafting rate of FA on the FA-BSA complex. The size distribution and zeta potentials were obtained using a Malven instrument. The samples were prepared at a concentration of 1 mg/mL, and the morphologies observed by TEM (HT-7700, Japan) and AFM (Nano IR2, Germany). The X-ray photoelectron spectroscopy (XPS) was used to analyze the chemical states by an S-Probe photoelectron spectrometer system (AXIS Supra, England). The stability of both MoOx and FA-BSA-PEG/MoOx was evaluated in DI water, PBS, normal saline and DMEM cell culture medium, respectively, and the nanosize and zeta potential were measured with different incubation times. The degradation of the nanosheet was also tested, and the detailed method described in Additional file 1.

##### In vitro photothermal properties

The temperature changes of the FA-BSA-PEG/MoOx solution with different concentrations (0, 20, 80, 100, 250, 500 and 1000 μg/mL) induced by NIR treatment were monitored under 1.5 W/cm^2^ irradiation. In addition, the temperature of the FA-BSA-PEG/MoOx was periodically recorded with a wavelength of 808 nm laser every 30 s. To evaluate the photothermal stability, the FA-BSA-PEG/MoOx solution with a concentration of 250 μg/mL was irradiated by the NIR laser for 0, 10, 20 and 30 min, and then cooled down to room temperature. Comparison the absorption spectrum of FA-BSA-PEG/MoOx nanosheet solution with/without laser irradiation. In addition, the test of repeated irradiation by the NIR laser for five rounds in 600 s intervals was also performed to evaluate the photothermal stability of the nanosheet. The photothermal conversion efficiency was calculated according to methods described in the literature [37].

##### In vitro drug loading and release

To investigate the drug loading capability of the nanosheet, DTX, the model drug, was loaded according to the reported literature [38]. Typically, the DTX and FA-BSA-PEG/MoOx solution were prepared at the concentration of 3 mg/mL, and mixtures of the two solutions with different ratios were prepared to optimize the formulation. Afterward, the DTX was determined by HPLC (the chromatographic conditions are shown in Additional file 1), and the drug loading and efficiency were calculated based on the following equation:

1 $${\text{EE}}\left( \% \right) = {\text{mass}}\;{\text{of}}\;{\text{DTX}}\;{\text{encapsulated}}\;{\text{in}}\;{\text{nanosheet}}/{\text{mass}}\;{\text{of}}\;{\text{feeding}}\;{\text{DTX}} \times 100\% ,$$

2 $${\text{DL}}\left( \% \right) = {\text{mass}}\;{\text{of}}\;{\text{DTX}}\;{\text{encapsulated}}\;{\text{in}}\;{\text{nanosheet}}/{\text{mass}}\;{\text{of}}\;{\text{DTX}}\;{\text{loaded}}\;{\text{polymer}} \times 100\% .$$

For the evaluation of DTX release behavior, 1 mL of the DTX loaded nanosheet (FA-BSA-PEG/MoOx@DTX) was transferred into a dialysis bag (3500 Da), which was placed in a 30 mL release medium (pH 5.0 containing 0.5% Tween 80). At the pre-designed time point (0.25, 0.5, 1, 2, 4, 6, 8, 12, 24, 36, 48 and 60 h), 1 mL of medium was taken out, and the same volume of fresh release medium was added. The concentration of the DTX was tested by the HPLC for release assay, and the assay conditions are described in Additional file 1.

##### In vitro assays

Hemolysis: The preparation of the diluted red blood cells (RBCs) was according to previously published work from our lab [39]. 1 mL of the normal saline containing different concentrations (0.01 to 1.0 mg mL^−1^) of nanosheet was taken out and mixed gently with 1 mL of the RBCs suspension, which was incubated for 1 h at 37 °C. The normal saline and DI water were set as the negative and positive control, respectively. At the end of the incubation, the absorbance of the supernatant at 540 nm was measured, and the hemolysis was calculated as follows:

3 $${\text{Percent}}\;{\text{hemolysis}}\left( \% \right) = \left( {{\text{Asample}} - {\text{Anegative}}\;{\text{control}}} \right)/\left( {{\text{Apositive}} - {\text{Anegative}}} \right) \times 100\% .$$

Cell viability: The cytotoxicities of the blank FA-BSA-PEG/MoOx and FA-BSA-PEG/MoOx@DTX nanosheets were evaluated using a CCK8 method. MCF-7 cells were seeded into 96-well plates at a density of 8000 cells/well and incubated for 24 h. Then, the old medium was aspirated, and 100 μL of blank medium (as the control) and different concentrations of free DTX, FA-BSA-PEG/MoOx and FA-BSA-PEG/MoOx@DTX was added and then incubated for 20 h. To investigate the effect of laser irradiation on the cell, the cell was treated by NIR laser (808 nm, 1.5 W/cm^2^) for 1 min and then incubated for 20 h. Finally, the CCK-8 kit was applied to evaluate cell viability.

Cell uptake assays: The coumarin-6 (C6) was applied for the fluorescence analysis. MCF-7 cells were seeded into a 12-well plate at a density of 1 × 10^5^ cells/well, and incubated for 24 h. Then, aspirate the old media, and 1 mL of FA-BSA-PEG/MoOx@C6 or BSA-PEG/MoOx@C6 nanosheets was added and incubated at a predetermined period (1, 2 and 4 h). Afterward, the cells were fixed by paraformaldehyde, and the cell nuclei were stained by DAPI according to the kit instructions, and the cell uptake was observed using a fluorescence microscope. Besides, the quantitative analysis was performed by flow cytometry.

Cell apoptosis assays: The MCF-7 cells were seeded in a 12-well plate (1 × 10^5^ cells/well) for 24 h. Then, aspirate the old media, and 1 mL of the fresh medium containing different concentrations of nanosheet was added. After incubation for 20 h, the cells were exposed to the NIR irradiation (1.5 W/cm^2^) for 10 min, and then incubated for another 4 h. The cells were then collected and stained by the Annexin V-FITC according to the kit instructions, and the cells were detected by flow cytometry.

Macrophage polarization: RAW 264.7 macrophages were cultured with IL-4 (25 ng/mL) for 12 h to induce M2 polarization. MCF-7 cells (1 × 10^5^/well) were seeded in a 12-well plate, respectively. After the cells adhered to 80% of the wall, the free DTX, FA-BSA-PEG/MoOx and FA-BSA-PEG/MoOx@DTX were added with or without NIR irradiation. The supernatant was then collected and used to incubate with M2 macrophages for another 12 h. Afterwards, RAW 264.7 macrophages were collected and stained by APC anti-CD86 and PE anti-CD206 antibodies, and then were measured by flow cytometry.

**ICD assays**

- Heat shock protein 70 (HSP 70): the MCF-7 cells were seeded into a cell culture dish (1 × 10^6^) overnight. Aspirate the old medium, and the fresh medium containing nanosheet was added. Then the cells were exposed to the NIR irradiation for 10 min and incubated for 20 h. Subsequently, the lysis buffer was added and the protein was collected for the Western blotting assay according to the protocol in our lab. The chemiluminescent (ECL) detection reagent was used to detect the protein, and the ChemiDoc MP imaging system (Bio-Rad) equipped with a chemiluminescence filter was used for analysis.
- CRT assays: the MCF-7 cells were cultured with the same protocol as above. After incubation for 24 h, cells were fixed with 4% paraformaldehyde for 30 min, incubated with rabbit anti-CRT antibody (dilution 1:100), and then the cells were stained with Dylight 488 goat anti-rabbit IgG (dilution 1:150) secondary antibody for 1 h. The nuclei were stained with DAPI. Images were obtained using confocal laser scanning microscopy (CLSM). Also, the CRT content was also tested by the Western blotting method.

#### In vivo assays

##### The biocompatibility of nanosheets in vivo

0.2 mL of FA-BSA-PEG/MoOx nanosheet solution was injected into healthy mice at a dose of 20 mg/kg. The normal saline was injected as the control, and the bodyweight of the mice was recorded. Then, the mice were sacrificed, and blood was collected in an anticoagulant-treated tube for analysis. The main organs (heart, liver, spleen, lung, kidney) were also retrieved for histological analysis and evaluation of the systemic toxicity.

##### Pharmacokinetic assays

The Wistar rats were randomly divided into two groups, and intravenously administered commercial Taxotere® and FA-BSA-PEG/MoOx@DTX at the dose of 10 mg/kg body weight. At the predetermined time points (5, 15, 30 min, 1, 3, 7, 12, 24, and 36 h), 0.4 mL of blood was collected and centrifuged at 3000 rpm for 10 min to collect plasma which was stored in a refrigerator at − 20 °C. The DTX concentration in the blood was determined by HPLC. The pharmacokinetic parameters were obtained from the DAS software.

##### Biodistribution assays

The single armpits subcutaneous 4T1 cell model was generated and the rats were randomly divided into two groups, and free IR780 solution or FA-BSA-PEG/MoOx@IR780 solution (the dose of IR780 is 10 mg/Kg) were intravenously administered. At pre-designed time points of 1, 4.5, 9, 12, and 24 h, the rat was observed in a small-animal imaging system, and the distribution was photographed and recorded. The mice were sacrificed, and the tumors and main organs were stripped off for fluorescence imaging at the 24 h time point.

##### Photothermal performance in vivo

The normal saline, FA-BSA-PEG/MoOx and FA-BSA-PEG/MoOx@DTX solutions were intravenously administered in mice at a dosage of 10 mg/kg. The dosage of FA-BSA-PEG/MoOx was equivalent to the dosage of nanosheets in FA-BSA-PEG/MoOx@DTX. The NIR treatment was added after administration of 24 h. As an alternative method, 40 μL of the FA-BSA-PEG/MoOx@DTX was also injected into the tumor directly, and the NIR irradiation (808 nm, 1 W/cm^2^, 10 min) was turned on immediately after injection. An infrared thermal imager was used for the imaging and temperature recording.

##### Anti-tumor effect

The tumor model generation and drug administration methods: To evaluate the effects of the treatment on primary and distant tumors, two tumor models were generated: a bilateral armpits subcutaneous 4T1 cell model and a single armpits subcutaneous 4T1 cell model. Then, the mice were treated with Taxotere® (DTX = 10 mg/kg), FA-BSA-PEG/MoOx@DTX (DTX = 10 mg/kg, with/without NIR treatment), FA-BSA-PEG/MoOx (with/without NIR treatment), all intravenously administered every 3 days, respectively. Changes in mouse tumors were recorded every 2 days.

The observation of the mice’s behaviors and tumor tissue: The body weight and both of the tumor volume of the mice were measured and recorded every 2 days, and the tumor inhibition rate was calculated. In addition, the samples were stained with immunofluorescence and hematoxylin and eosin (H&E) dyes and imaged. The images were then used for the tumor tissue analysis.

**Immune response in vivo**

- DCs mature: the inguinal lymph nodes were collected, and DCs were analyzed by flow cytometry. Mature DCs were defined as CD11c^+^, CD80^+^ and CD86^+^.
- T cell infiltration (in tumor and spleen tissue): the tumors and spleen tissues were collected, and the percentages of CTLs (CD3^+^, CD8^+^) and Th cells (CD3^+^, CD4^+^) were determined and analyzed by flow cytometry.
- Cytokines: the blood was collected after treatment by different formulations, and tumor necrosis factor-α (TNF-α), interferon-γ (IFN-γ), interleukin 2 (IL-2) and interleukin 6 (IL-6) were detected using ELISA kits.

Anti-lung metastatic evaluation: After the single tumor was generated, 4T1 cells (8 × 10^5^) were injected through the tail vein. The mice were then randomly divided into 4 groups (n = 6) and were treated with normal saline, Taxotere® (DTX = 10 mg/kg) and FA-BSA-PEG/MoOx@DTX (DTX = 10 mg/kg) (with or without NIR treatment) every 3 days. The lungs of the mice were taken out and photographed on the 13th day after 4 times of treatments. The other organs (heart, liver, spleen, kidney) and tumor were retrieved for H&E analysis.

#### Statistic

Significant differences were evaluated using SPSS software. All experiments were repeated at least three times. The difference was considered significant when *p* < 0.05 (*), *p* < 0.01 (**), *p* < 0.001 (***).

## Supplementary Information

**Additional file 1: Figure S1.** FTIR spectrum of FA-BSA. **Figure S2.** The magnification of a part of FA-BSA-PEG/MoOx spectrum. **Figure S3.** (A) UV spectrum of FA; (B) standard curve of FA; (C) standard curve of BSA; (D) UV absorption spectrum of a solution of FA and BSA. **Figure S4.** UV-Vis absorption spectra of MoOx and FA-BSA-PEG/MoOx nanosheets. **Figure S5.** The thickness of (A) MoOx and (B) FA-BSA-PEG/MoOx nanosheets. **Figure S6.** UV absorption spectra of FA-BSA-PEG/MoOx nanosheets after different times of irradiation. **Figure S7.** Infrared thermal images of FA-BSA-PEG/MoOx nanosheets with different concentration and irradiation time periods. **Figure S8.** Degradation profile of the nanosheets based on the UV-Vis absorption spectra of FA-BSA-PEG/MoOx nanosheets incubated in different PBS solutions at different time points. **Figure S9.** UV Vis absorption spectra of FA-BSA-PEG/MoOx nanosheets incubated in (A) pH 5.0 and (B) pH 7.4 serum for different time periods. **Figure S10.** HPLC chromatogram of DTX. **Figure S11.** The release curve of FA-BSA-PEG/MoOx@DTX in PBS buffer with pH 5.0. **Figure S12.** The flow cytometry analysis of FA-BSA-PEG/MoOx@C6 nanosheets uptake by MCF-7 cells at 1, 2, 4 h. **Figure S13.** (A)The western blot analysis of CRT and (B) CRT/β-actin ratio after various treatments. **Figure S14.** The biochemical indexes of tumor-bearing mice after different treatments : (A) ALT;(B) AST;(C) BUN and (D) CR, respectively. **Figure S15.** (A) Infrared thermal images of tumor-bearing mice and (B)Temperature-change curves of the tumor being irradiated after intratumoral injection. **Figure S16.** The tumor volume in mice treated by different formulations. **Figure S17.** H&E staining pictures of organs and tumors of mice after different treatments, scale bar: 200 μm. **Table S1.** Drug loading of nano tablets in FA-BSA-PEG/MoOx nanosheets (n = 3). **Table S2.** The hemolysis of HBRCs treated with FA-BSA-PEG/MoOx nanosheets at different concentrations. **Table S3.** Blood analysis of mice on the 15th day post-injection of FA-BSA-PEG/MoOx (20 mg/kg) or saline in the tail vein. **Table S4.** DTX concentration in blood at different time points after intravenous administration commercial Taxotere and FA-BSA-PEG/MoOx@DTX nanosheets in rats (n = 3).

## Data Availability

All data generated or analyzed during this study are included in this manuscript and its additional files.
