# Supplementary material for: A molybdenum oxide-based degradable nanosheet for combined chemo-photothermal therapy to improve tumor immunosuppression and suppress distant tumors and lung metastases
Source: J Nanobiotechnology. 2021 Dec 19;19:428. doi: 10.1186/s12951-021-01162-2 (PMC8684628; doi:10.1186/s12951-021-01162-2)
Supplement: Supplementary file 1 — Additional file 1: Figure S1. FTIR spectrum of FA-BSA. Figure S2. The magnification of a part of FA-BSA-PEG/MoOx spectrum. Figure S3. (A) UV spectrum of FA; (B) standard curve of FA; (C) standard curve of BSA; (D) UV absorption spectrum of a solution of FA and BSA. Figure S4. UV-Vis absorption spectra of MoOx and FA-BSA-PEG/MoOx nanosheets. Figure S5. The thickness of (A) MoOx and (B) FA-BSA-PEG/MoOx nanosheets. Figure S6. UV absorption spectra of FA-BSA-PEG/MoOx nanosheets after different times of irradiation. Figure S7. Infrared thermal images of FA-BSA-PEG/MoOx nanosheets with different concentration and irradiation time periods. Figure S8. Degradation profile of the nanosheets based on the UV-Vis absorption spectra of FA-BSA-PEG/MoOx nanosheets incubated in different PBS solutions at different time points. Figure S9. UV Vis absorption spectra of FA-BSA-PEG/MoOx nanosheets incubated in (A) pH 5.0 and (B) pH 7.4 serum for different time periods. Figure S10. HPLC chromatogram of DTX. Figure S11. The release curve of FA-BSA-PEG/MoOx@DTX in PBS buffer with pH 5.0. Figure S12. The flow cytometry analysis of FA-BSA-PEG/MoOx@C6 nanosheets uptake by MCF-7 cells at 1, 2, 4 h. Figure S13. (A)The western blot analysis of CRT and (B) CRT/β-actin ratio after various treatments. Figure S14. The biochemical indexes of tumor-bearing mice after different treatments : (A) ALT;(B) AST;(C) BUN and (D) CR, respectively. Figure S15. (A) Infrared thermal images of tumor-bearing mice and (B)Temperature-change curves of the tumor being irradiated after intratumoral injection. Figure S16. The tumor volume in mice treated by different formulations. Figure S17. H&E staining pictures of organs and tumors of mice after different treatments, scale bar: 200 μm. Table S1. Drug loading of nano tablets in FA-BSA-PEG/MoOx nanosheets (n = 3). Table S2. The hemolysis of HBRCs treated with FA-BSA-PEG/MoOx nanosheets at different concentrations. Table S3. Blood analysis of mice on the 15th day post-inj [file 12951_2021_1162_MOESM1_ESM.docx]

**Additional file**

**A Molybdenum Oxide-based Degradable Nanosheet for Combined Chemo-photothermal Therapy to Improve** **Tumor Immunosuppression and** **Suppress Distant Tumors and Lung Metastases**

Na Qiu^1^, Xiaoye Yang^1^, Yanan Zhang^1^, Jicheng Zhang ^2^, Jianbo Ji^1^, Yu Zhang^1^, Xinru Kong^1^, Yanwei Xi^1^, Dongzhu Liu^1^, Lei Ye^1^*, Guangxi Zhai^1^*

*^1^Department of Pharmaceutics, Key Laboratory of Chemical Biology (Ministry of Education), School of Pharmaceutical Sciences, Shandong University, Jinan, 250012, P. R. China*

*^2^Department of Chemistry and Bioengineering, University of Washington, Seattle, WA, 98195, USA*

**1. The degradation assay**

The degradation behavior of the nanosheet was investigated in PBS solution and serum (40%). The FA-BSA-PEG/MoOx nanosheets were dispersed in PBS buffer with different pH values (pH= 3.4, 5.0, 6.5, 7.4, 9.4 and 11.4). At predetermined time intervals, 5 mL of the buffer was taken out, and 5ml of fresh medium was added. The retrieved buffer was scanned by the UV and digital photos were taken. The absorbance value at 808 nm was used to calculate the degradation speed. In addition, the nanosheets were incubated with PBS at pH 5.0, 7.4, and 11.4 for 2 h at room temperature, and then, the morphologies of the nanosheets were studied using TEM. To stimulate *in vivo* environment, the degradation was also investigated in 40% serum under the same conditions.

**2. Chromatographic method for HPLC assay**

DTX was determined using HPLC: Column: HyperSil-ODS2 reverse phase chromatography column (4.6×250 mm, 5 μm); detection wavelength: 231 nm; mobile phase: acetonitrile: distilled water = 55: 45 (v / v); flow rate: 1.0 mL/min; The injection volume was 20 μL.





Figure S1 FTIR spectrum of FA-BSA.


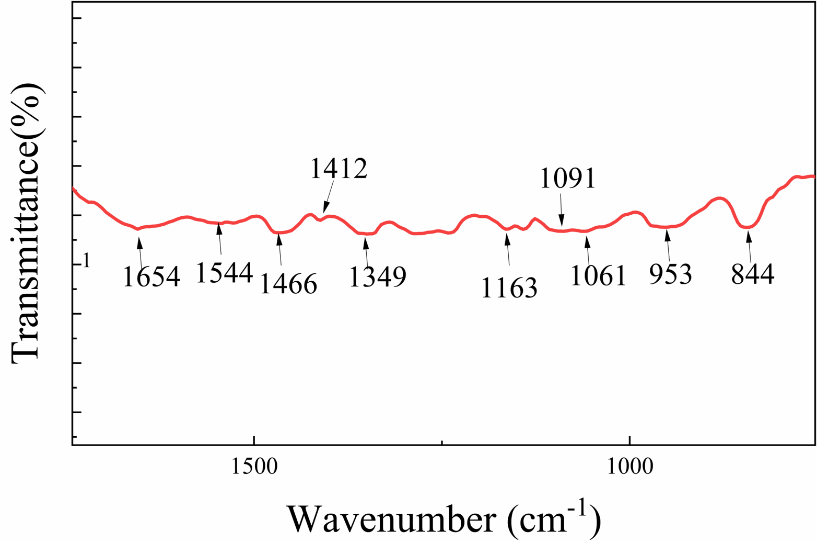


Figure S2 The magnification of a part of FA-BSA-PEG/MoOx spectrum.


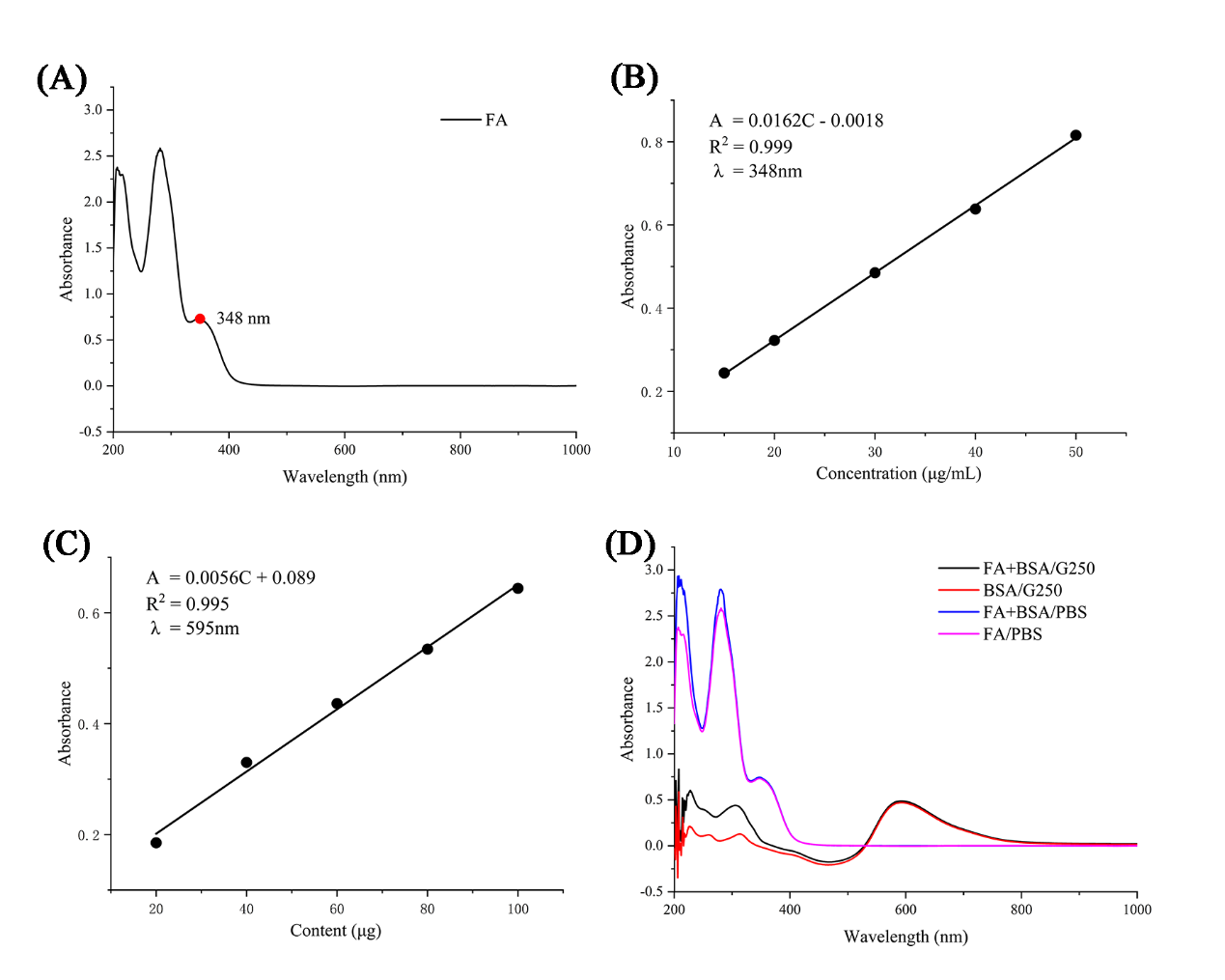


Figure S3 (A) UV spectrum of FA; (B) standard curve of FA; (C) standard curve of BSA; (D) UV absorption spectrum of a solution of FA and BSA.





Figure S4 UV-Vis absorption spectra of MoOx and FA-BSA-PEG/MoOx nanosheets.


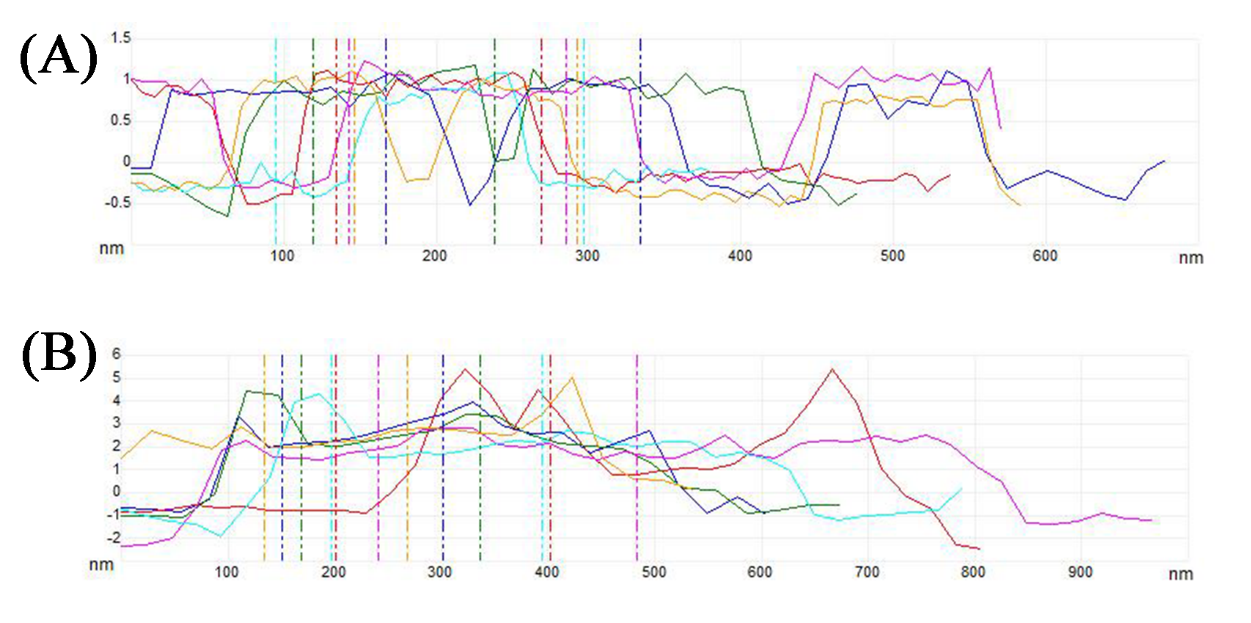


Figure S5 The thickness of (A) MoOx and (B) FA-BSA-PEG/MoOx nanosheets.





Figure S6 UV absorption spectra of FA-BSA-PEG/MoOx nanosheets after different times of irradiation.


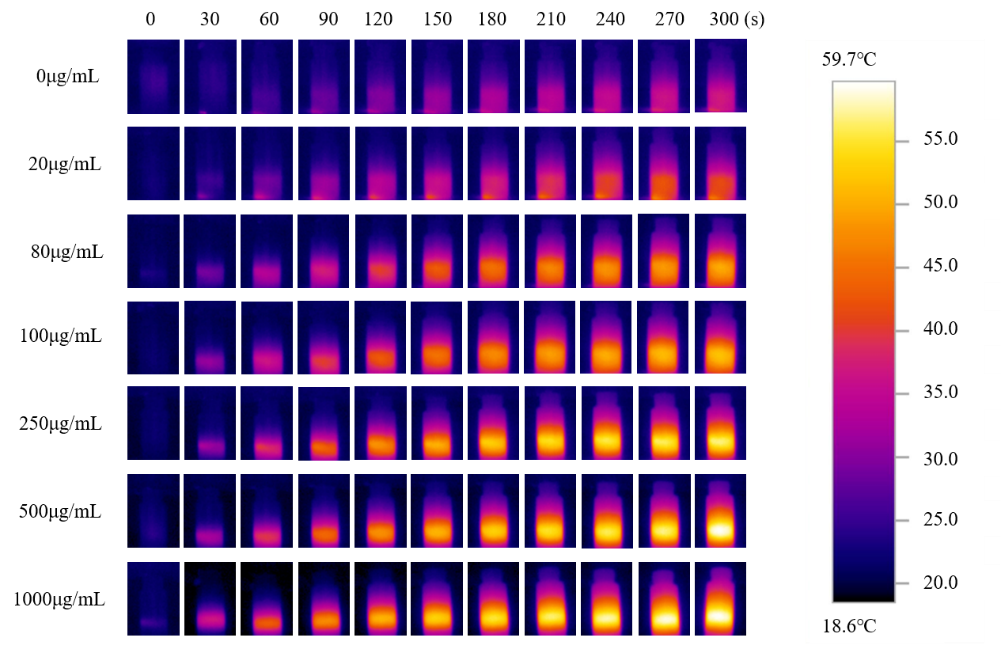


Figure S7 Infrared thermal images of FA-BSA-PEG/MoOx nanosheets with different concentration and irradiation time periods.


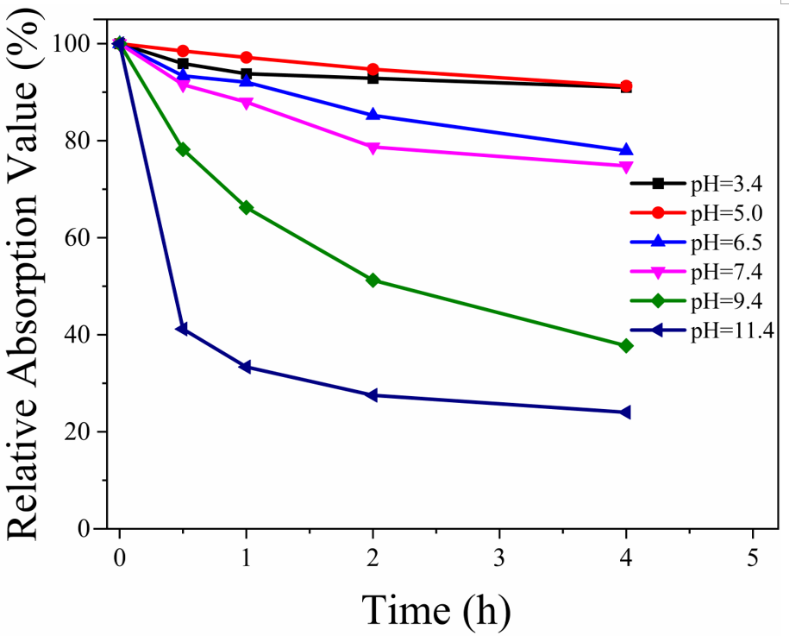


Figure S8 Degradation profile of the nanosheets based on the UV-Vis absorption spectra of FA-BSA-PEG/MoOx nanosheets incubated in different PBS solutions at different time points.


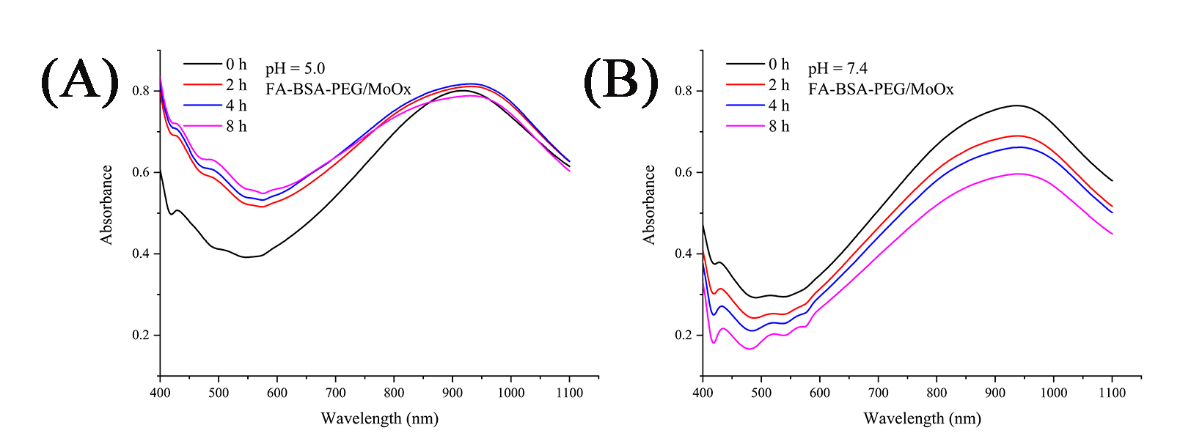


Figure S9 UV Vis absorption spectra of FA-BSA-PEG/MoOx nanosheets incubated in (A) pH 5.0 and (B) pH 7.4 serum for different time periods.

Figure S10 HPLC chromatogram of DTX.





Figure S11 The release curve of FA-BSA-PEG/MoOx@DTX in PBS buffer with pH 5.0.


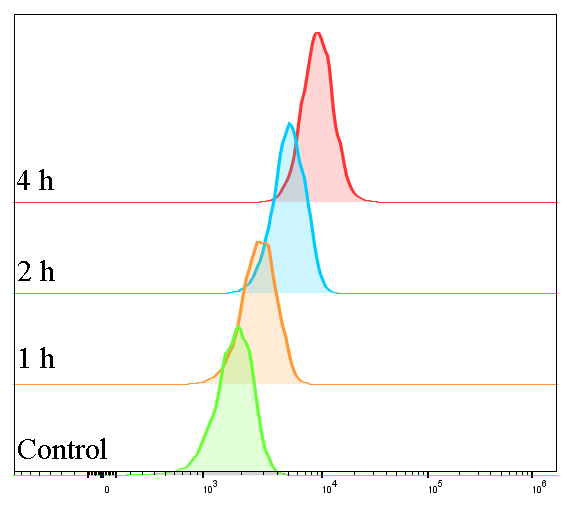


Figure S12 The flow cytometry analysis of FA-BSA-PEG/MoOx@C6 nanosheets uptake by MCF-7 cells at 1, 2, 4 h.


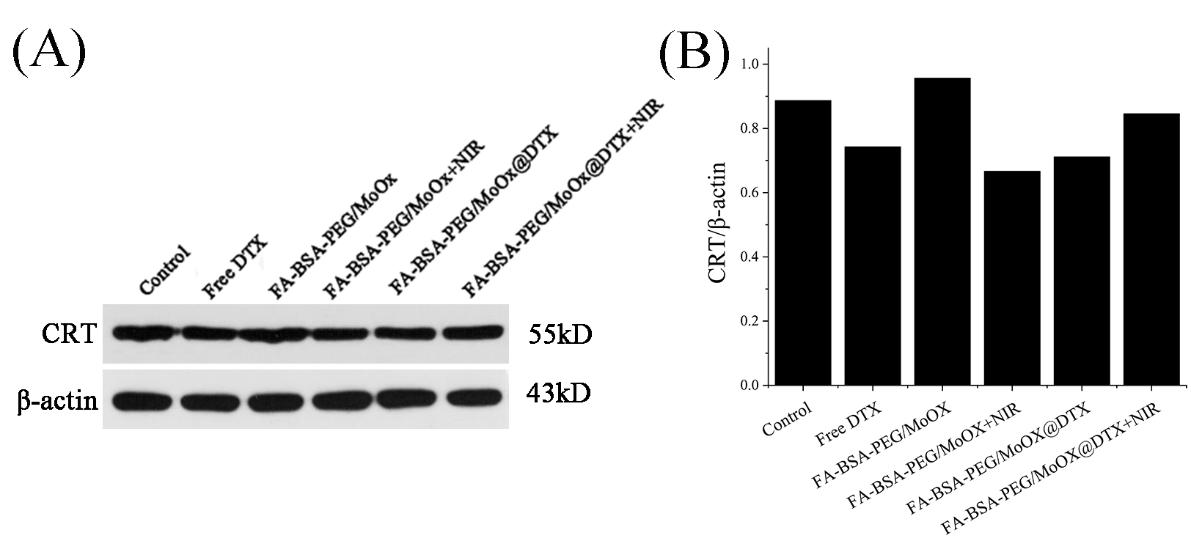


Figure S13 (A)The western blot analysis of CRT and (B) CRT/β-actin ratio after various treatments.


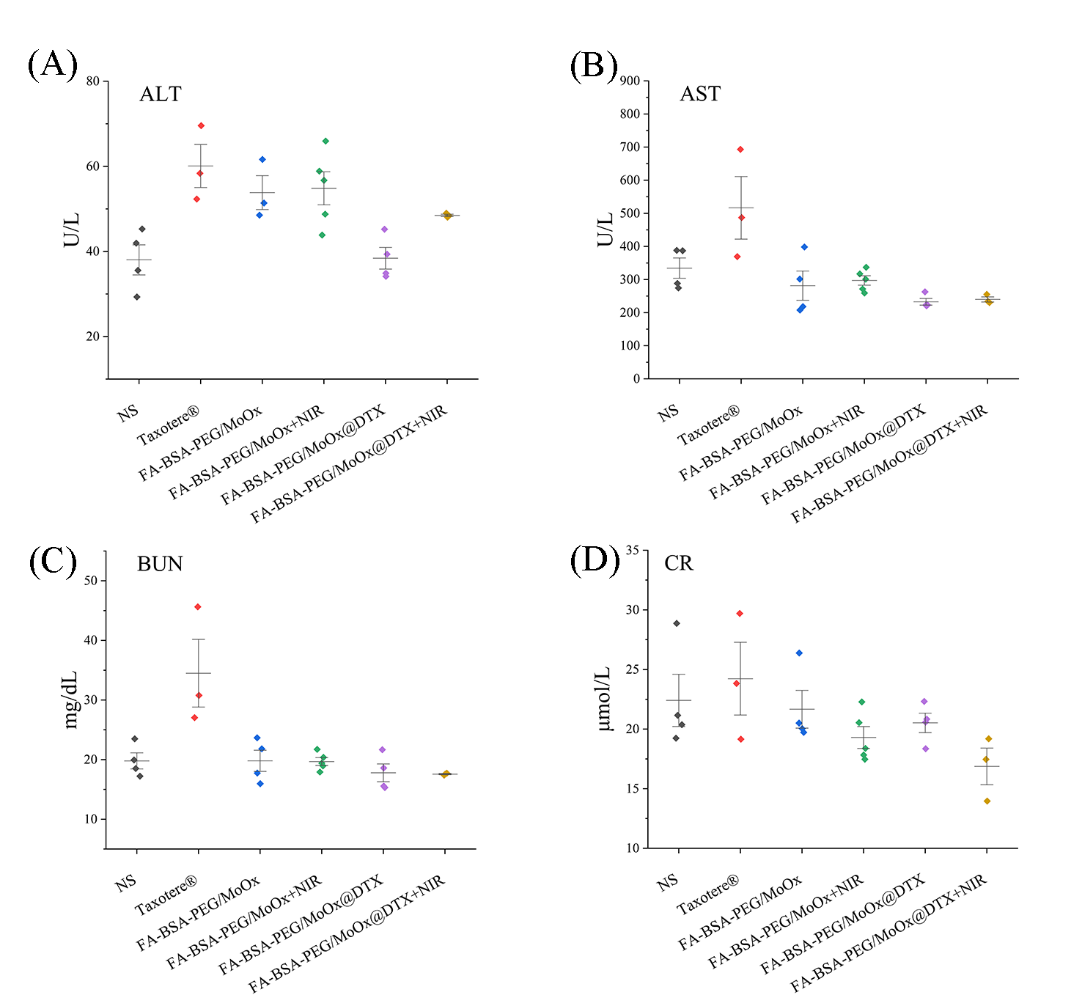


Figure S14 The biochemical indexes of tumor-bearing mice after different treatments : (A) ALT;(B) AST;(C) BUN and (D) CR, respectively.


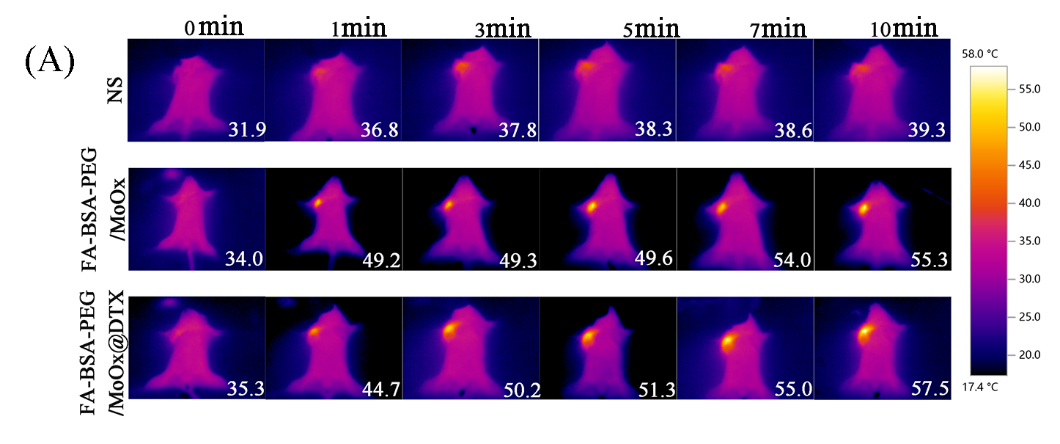


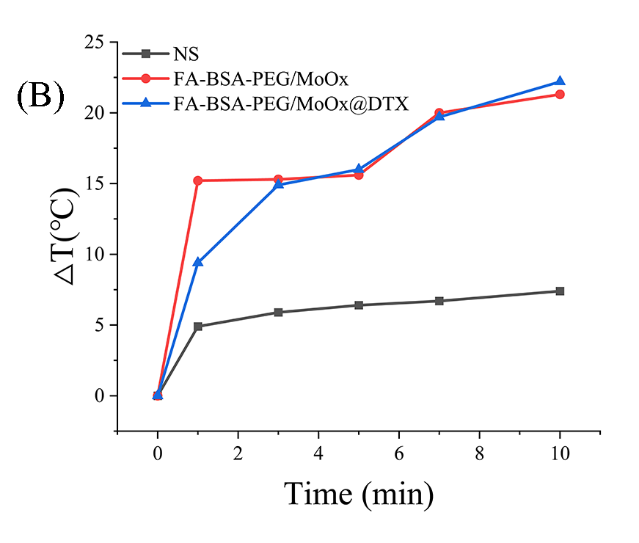


Figure S15 (A) Infrared thermal images of tumor-bearing mice and (B)Temperature-change curves of the tumor being irradiated after intratumoral injection.


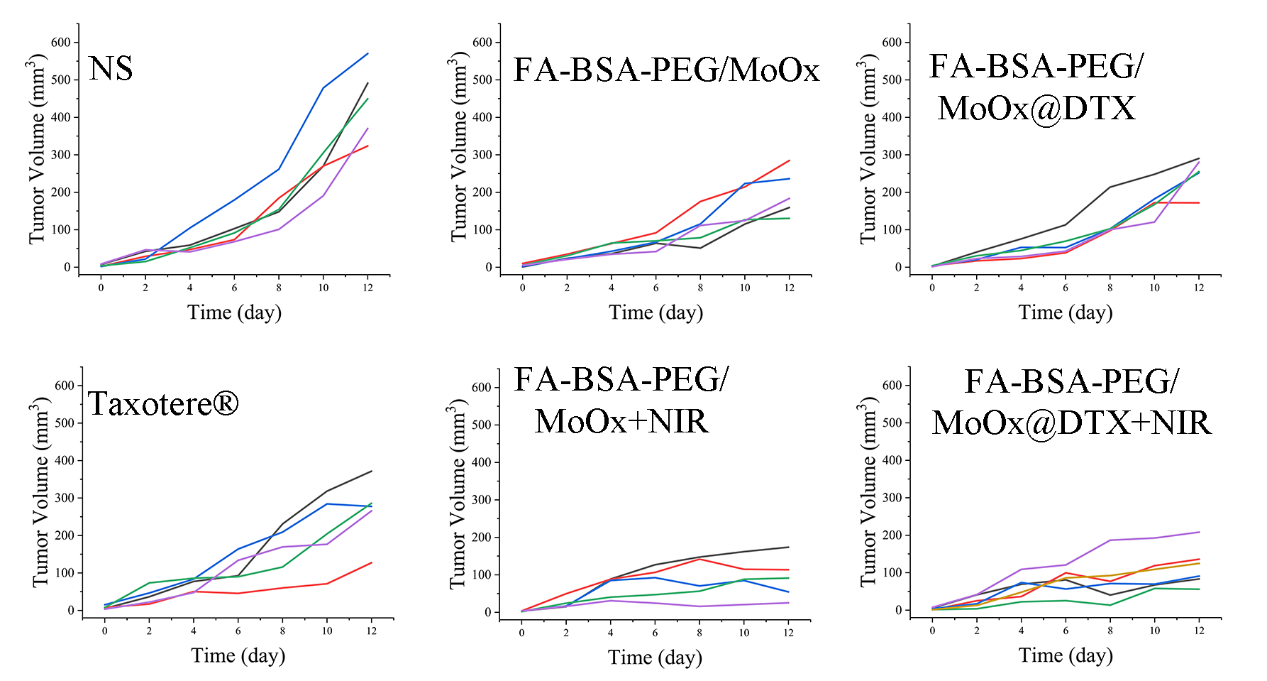


Figure S16 The tumor volume in mice treated by different formulations.


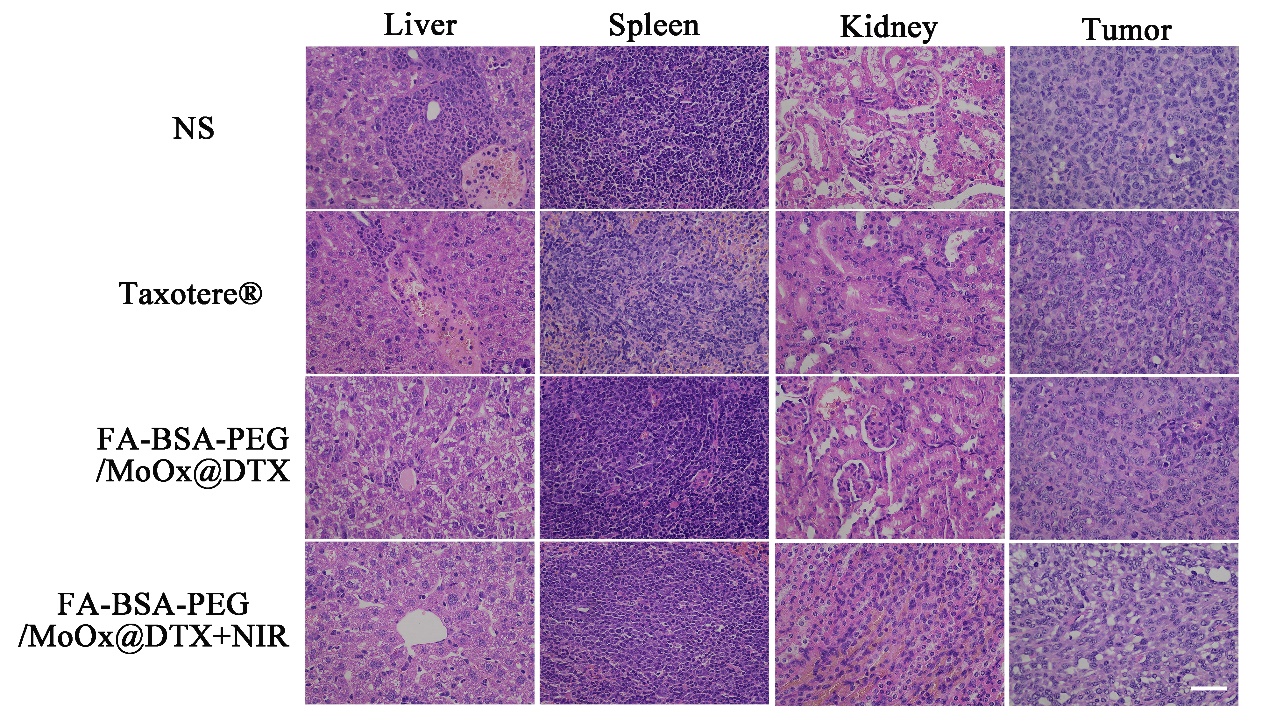


Figure S17 H&E staining pictures of organs and tumors of mice after different treatments, scale bar: 200 μm.

Table S1 Drug loading of nano tablets in FA-BSA-PEG/MoOx nanosheets (n = 3)

| Feeding ratio (m/m) | Drug loading (%) | Encapsulation rate (%) |
| --- | --- | --- |
| 1:1 | 45.61 ± 0.81 | 83.88 |
| 1:2 | 62.78 ± 0.06 | 84.12 |
| 1:4 | 76.49 ± 0.04 | 81.33 |

Table S2 The hemolysis of HBRCs treated with FA-BSA-PEG/MoOx nanosheets at different concentrations

| Concentration（mg·mL^−1^） | 0.01 | 0.02 | 0.05 | 0.1 | 0.5 | 1.0 |
| --- | --- | --- | --- | --- | --- | --- |
| hemolysis（%） | 0.40 ± 0.40 | 0.81 ± 0.22 | 0.63 ± 0.47 | 0.96 ± 0.15 | 1.46 ± 0.24 | 2.54 ± 0.81 |

Table S3 Blood analysis of mice on the 15^th^ day post-injection of FA-BSA-PEG/MoOx (20 mg/kg) or saline in the tail vein.

| **Parameters** | **Abbreviation** | **Saline** | **Nanosheet** | **Unit** | **Conference** |
| --- | --- | --- | --- | --- | --- |
| White cells | WBC | 2.0±1.07 | 4.16±2.45 | 10^9^/L | 0.8-6.8 |
| Lymphocytes | Lymph# | 1.33±0.61 | 2.97±1.81 | 10^9^/L | 0.7-5.7 |
| Monocyte | Mon# | 0.07±0.06 | 0.14±0.11 | 10^9^/L | 0.0-0.3 |
| Neutrophils | Gran# | 0.63±0.42 | 1.04±0.56 | 10^9^/L | 0.1-1.8 |
| Lymphocytes | Lymph% | 64.13±3.26 | 69.23±6.10 | % | 55.8-90.6 |
| Monocyte (%) | Mon% | 3.63±1.24 | 3.40±0.94 | % | 1.8-6.0 |
| Neutrophils (%) | Gran% | 32.23±2.75 | 27.37±5.89 | % | 8.6-38.9 |
| Red cells | RBC | 8.56±2.13 | 9.27±2.28 | 10^12^/L | 6.36-9.42 |
| Hemoglobin | HGB | 115±19 | 134.14±31.90 | g/L | 110-143 |
| Red blood cell backlog | HCT | 42.3±10.44 | 46.53±10.49 | % | 34.6-46.6 |
| Mean red blood cell volume | MCV | 49.53±0.81 | 50.5±1.55 | fL | 48.2-58.3 |
| Average content of red blood cell hemoglobin | MCH | 13.63±1.65 | 14.44±0.38 | pg | 15.8-19 |
| Mean concentration of red blood cell hemoglobin | MCHC | 276.76±34.70 | 287±6.38 | g/L | 302-353 |
| Coefficient of variation of red blood cell distribution width | RDW | 17.7±0.62 | 17.16±1.05 | % | 13-17 |
| Platelet | PLT | 495±204.87 | 1031± 71.46 | 10^9^/L | 450-1590 |
| Mean platelet volume | MPV | 6.83±0.23 | 5.73±0.55 | fL | 3.8-6.0 |
| Platelet distribution width | PDW | 17.17±0.40 | 16.63±0.59 |  |  |
| Platelet packed volume | PCT | 0.34±0.13 | 0.41±0.19 | % |  |

Table S4 DTX concentration in blood at different time points after intravenous administration commercial Taxotere and FA-BSA-PEG/MoOx@DTX nanosheets in rats (n = 3)

| Time (h) | DTX concentration (μg/mL) | |
| --- | --- | --- |
|  | Taxotere | FA-BSA-PEG/MoOx@DTX |
| 0.083 | 8.37±1.32 | 17.03±2.06 |
| 0.25 | 1.73±0.17 | 13.08±1.04 |
| 0.5 | 0.54±0.19 | 4.93±0.21 |
| 1 | 0.25±0.03 | 2.12±0.05 |
| 2 | 0.19±0.03 | 0.51±0.10 |
| 4 | 0.17±0.01 | 0.25±0.06 |
| 6 | 0.12±0.70 | 0.23±0.05 |
| 8 | 0.10±0.06 | 0.15±0.01 |
| 12 | - | 0.13±0.03 |
| 24 | - | 0.12±0.01 |
| 36 | - | 0.09±0.01 |
